# Combined Effects of *Chlamydia pneumoniae* Infection and Hypoxia on Glial Metabolism and Alzheimer’s Disease-Related Pathways

**DOI:** 10.3390/ijms27198820

**Published:** 2026-10-02

**Authors:** Kabita Kuikel, Amy McEwen, Tahlia Joy Mathiske, Lucinda Wojtaszak, James A. St John, Ali Delbaz

**Affiliations:** Clem Jones Centre for Neurobiology and Stem Cell Research, Institute for Biomedicine and Glycomics, Griffith University, Gold Coast, QLD 4215, Australia

**Keywords:** glial metabolism, Alzheimer’s disease, hypoxia, amyloid-β, dual stress response, glial immunometabolism, *Chlamydia pneumoniae*

## Abstract

Alzheimer’s disease involves complex interactions among neuroinflammation and metabolic dysfunction. Emerging evidence implicates microbial pathogens and hypoxia as converging stressors, yet their combined impact on glial function remains poorly understood. Human astrocyte and microglial cell lines were exposed to four experimental conditions: (i) normoxic control, (ii) *Chlamydia pneumoniae* infection under normoxia, (iii) hypoxia control and (iv) *Chlamydia pneumoniae* infection under hypoxic conditions. Hypoxia was induced at 3% oxygen for 72 h. Cellular responses were assessed using NanoString nCounter transcriptomics using the Alzheimer’s Disease and Metabolic Pathways panels, Tandem Mass Tag proteomics, MTT reduction, glucose uptake, intracellular ATP quantification and amyloid beta quantification by ELISA. Combined exposure to infection and hypoxia was associated with cell-type-specific alterations in glial metabolic and transcriptional responses. Factorial transcriptomic analysis identified significant infection × oxygen interactions across both NanoString panels and glial cell types. Proteomic responses differed according to cell type and oxygen availability. Aβ40 and Aβ42 measurements demonstrated treatment-associated alterations in the extracellular Aβ peptide profile, including increased Aβ42/Aβ40 ratios following infection. Together, these findings demonstrate distinct and interacting effects of infection and hypoxia on glial metabolic and molecular responses and identify Alzheimer’s disease-related cellular changes warranting further investigation in physiologically relevant models.

## 1. Introduction

Alzheimer’s disease (AD) is the leading cause of dementia worldwide, affecting more than 55 million people, and its prevalence is projected to increase substantially as global populations continue to age [1]. Neuropathologically, AD is defined by hallmark lesions: extracellular amyloid-β (Aβ) plaques [2,3,4], intracellular aggregates of hyperphosphorylated tau in neurofibrillary tangles [5,6], synaptic dysfunction [5,7] and progressive neuronal loss [8,9,10]. Although advancing age and genetic predisposition, particularly the presence of the APOE ε4 allele, are well-established risk factors for AD, these factors alone do not fully account for disease onset, progression variability, or the marked clinical heterogeneity observed among individuals [11,12,13]. Increasing evidence indicates that AD arises from the combined influence of chronic neuroinflammation, metabolic dysregulation and vascular compromise. These pathogenic processes interact to destabilize neuronal and glial networks and accelerate neurodegeneration [14,15,16]. Understanding how these mechanisms converge is essential for identifying targets that can be developed into effective strategies for therapy and prevention.

Among alternative hypotheses for AD etiology, microbial involvement has earned increasing attention. The “infectious hypothesis” suggests that pathogens accessing the central nervous system (CNS) trigger innate immune responses, sustain neuroinflammation and promote protein misfolding and aggregation [17,18,19,20,21]. Several infectious agents, including Herpes simplex virus type 1, *Porphyromonas gingivalis* and fungi have been associated with AD through convergent evidence from epidemiological studies, post-mortem brain analyses and experimental research [21,22,23,24,25]. One influential idea proposes that Aβ itself functions as an antimicrobial peptide, trapping pathogens but unintentionally contributing to plaque formation [18,26]. Within this framework, *Chlamydia pneumoniae* (*C. pneumoniae*), an obligate intracellular bacterium associated with chronic respiratory infections, has emerged as a particularly compelling candidate. First reported in AD brain tissue, *C. pneumoniae* has since been repeatedly detected in vulnerable regions such as the hippocampus and entorhinal cortex [27]. Animal studies show that intranasal inoculation allows *C. pneumoniae* to traverse the olfactory nerve pathways, access the CNS and induce AD-like neuropathology, although human research remains controversial [28,29].

Glial cells are increasingly recognized as active contributors to AD pathogenesis. Microglia, the brain’s resident immune cells, normally support homeostasis through surveillance and debris clearance. In AD, they transition to disease-associated phenotypes, producing high levels of cytokines (IL-1β, TNF-α, IL-6) and generating excess reactive oxygen species (ROS), which exacerbate neuroinflammation, reduce phagocytic capacity and drive profound alterations in cellular metabolism [30,31,32,33,34,35,36]. Astrocytes, which normally sustain neuronal metabolism via lactate shuttling and provide trophic support, also become reactive and lose homeostatic functions, often developing neurotoxic phenotypes [37,38,39,40,41]. Single-cell transcriptomic studies consistently identify disease-associated microglia and reactive astrocyte populations that are enriched in AD brains, highlighting glial dysfunction as a central driver of disease progression [32,42,43,44].

A key yet underexplored aspect of this glial transformation is metabolic reprogramming. Upon activation, microglia frequently shift from oxidative phosphorylation (OXPHOS) to glycolysis, a Warburg-like state that fuels inflammatory signalling while impairing reparative functions [45,46,47,48,49]. Astrocytic mitochondrial dysfunction further damages the astrocyte–neuron lactate shuttle, depriving neurons of metabolic support and increasing oxidative stress [40,41,50]. Hypoxia, common in ageing and in AD brains due to vascular insufficiency and cerebral hypoperfusion, amplifies these processes by stabilizing hypoxia-inducible factor 1α (HIF-1α), which promotes glycolysis, alters inflammatory gene expression and boosts amyloidogenic APP processing [51,52,53]. Recent reviews consistently conclude that hypoxia exacerbates Aβ accumulation, tau pathology and glial dysfunction, thereby providing a mechanistic link between vascular impairment and neurodegeneration [54,55,56,57,58].

Emerging data now link *C. pneumoniae* infection to these metabolic and hypoxic factors. *C. pneumoniae* extensively reprograms host cell energy processes, hijacking mitochondria to gather nutrients and sustain persistence, especially under low-oxygen conditions where host glycolysis supports bacterial growth [59,60,61]. Notably, *C. pneumoniae* influences HIF-1α in a biphasic way: stabilizing HIF-1α early to boost glucose uptake but later degrading it via the secreted protease *Chlamydial* Protease-Like Activity Factor (CPAF) to keep a permissive environment [62,63,64]. This dual-phase regulation shows the bacterium’s ability to exploit and then undermine host hypoxia pathways. Beyond infection biology, recent transcriptomic studies in myeloid cells reveal that hypoxia and inflammatory signals combine to create distinct transcriptional responses—“combined-stressor” transcriptional states not seen with either stressor alone [65,66,67]. These insights are consistent with the pathological brain environment, where infection and vascular dysfunction may converge to drive distinct glial activation states.

Based on this body of evidence, we hypothesize that *C. pneumoniae* infection reprograms human astrocytes and microglia toward distinct immunometabolic states and that hypoxia amplifies this reprogramming under combined stress conditions.

We predict that combined microbial and hypoxic stress promotes alterations in glucose metabolism, accompanied by lineage-specific effects on cellular energetics, with distinct responses in astrocytes and microglia. This metabolic remodelling is expected to coincide with widespread changes in innate immune, mitochondrial, lipid and proteostatic pathways and with alterations in the extracellular amyloid-β peptide profile consistent with the antimicrobial protection model of Aβ. Based on previous evidence, *C. pneumoniae* and hypoxia may converge on mitochondrial and hypoxia-responsive metabolic pathways to influence glial activation and amyloid-β-associated processes [41,48,68,69].

To test this hypothesis, we exposed human astrocyte and microglia cell lines to four conditions: (i) normoxic control, (ii) *C. pneumoniae* infection under normoxia, (iii) hypoxia control and (iv) *C. pneumoniae* infection under hypoxic conditions and performed multiple readouts to assess transcriptional, proteomic, metabolic and Aβ responses. This factorial approach was designed to investigate the individual and combined effects of infection and hypoxia across molecular and functional outcomes.

By explaining how microbial and vascular stressors such as hypoxia converge to alter glial states, this work aims to refine the infectious hypothesis of AD within the context of an aging, hypoperfused brain. Our findings may help identify testable biomarkers at the intersection of infection, hypoxia and glial metabolism and inform future strategies targeting glial metabolic dysfunction.

## 2. Results

### 2.1. C. pneumoniae Localisation Under Normoxia and Hypoxia

**Figure 1:**
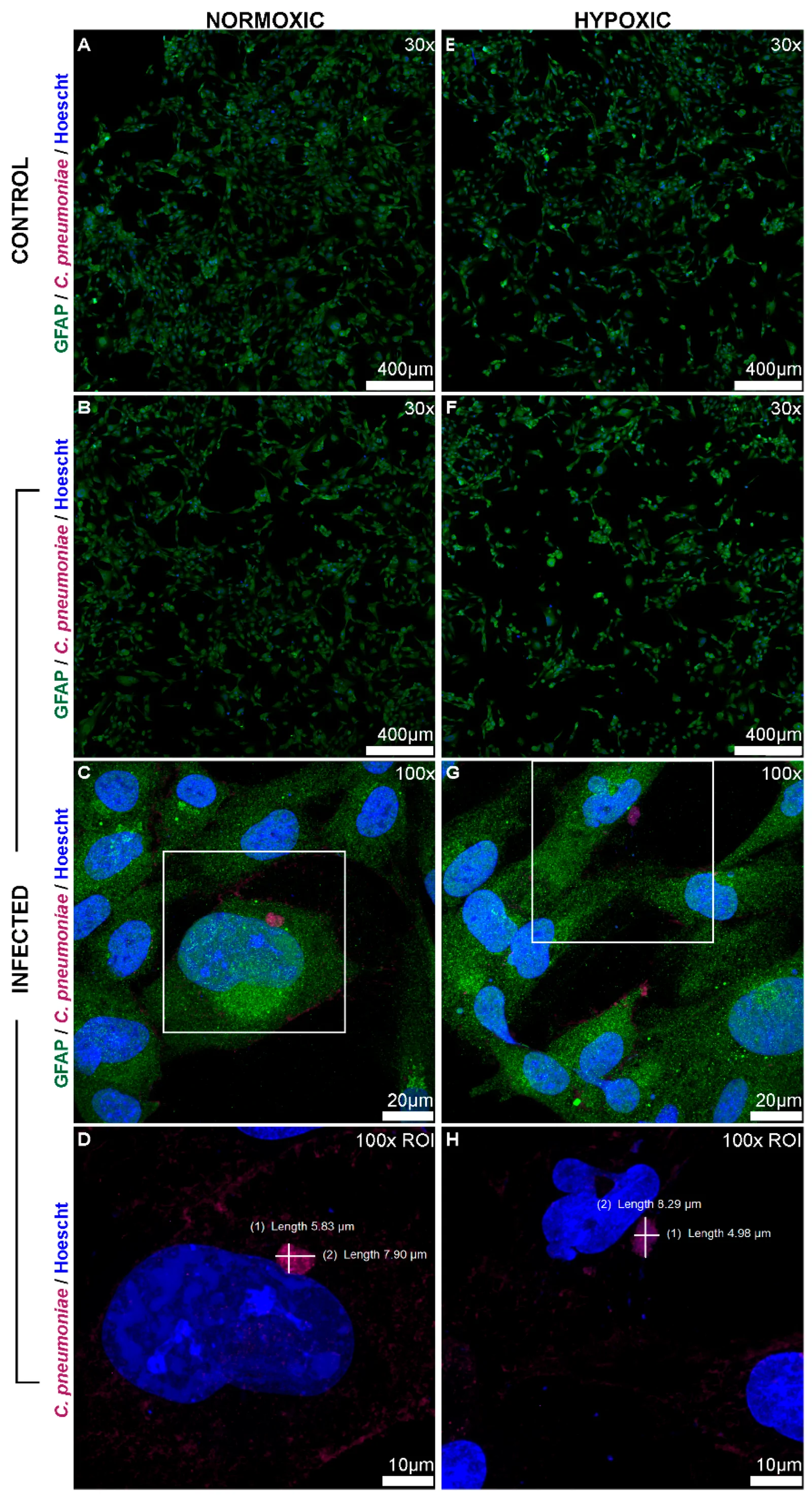
Immunofluorescence confirmation of astrocyte phenotype and *C. pneumoniae* infection under normoxic and hypoxic conditions. Human SVG p12 astrocytes were maintained under normoxic or hypoxic (3% O_2_) conditions and either left uninfected (control) or infected with *C. pneumoniae* (MOI = 1) for 72 h. (**A**,**E**) Representative 30× images of control astrocytes under normoxic (**A**) and hypoxic (**E**) conditions. (**B**,**F**) Representative 30× images of *C. pneumoniae*-infected astrocytes under normoxic (**B**) and hypoxic (**F**) conditions. (**C**,**G**) Representative 100× images of infected astrocytes under normoxic (**C**) and hypoxic (**G**) conditions, demonstrating intracellular *C. pneumoniae* immunoreactivity. White boxes indicate regions of interest shown as cropped enlargements in (**D**) and (**H**), respectively. (**D**,**H**) Cropped enlargements of the indicated regions from the corresponding 100× images, highlighting intracellular *C. pneumoniae*-positive structures and their measured dimensions. Astrocytes were labelled for GFAP (green), *C. pneumoniae* LPS (magenta) and nuclei with Hoechst (blue). Scale bars = 400 µm (**A**,**B**,**E**,**F**), 20 µm (**C**,**G**) and 10 µm (**D**,**H**).

**Figure 2:**
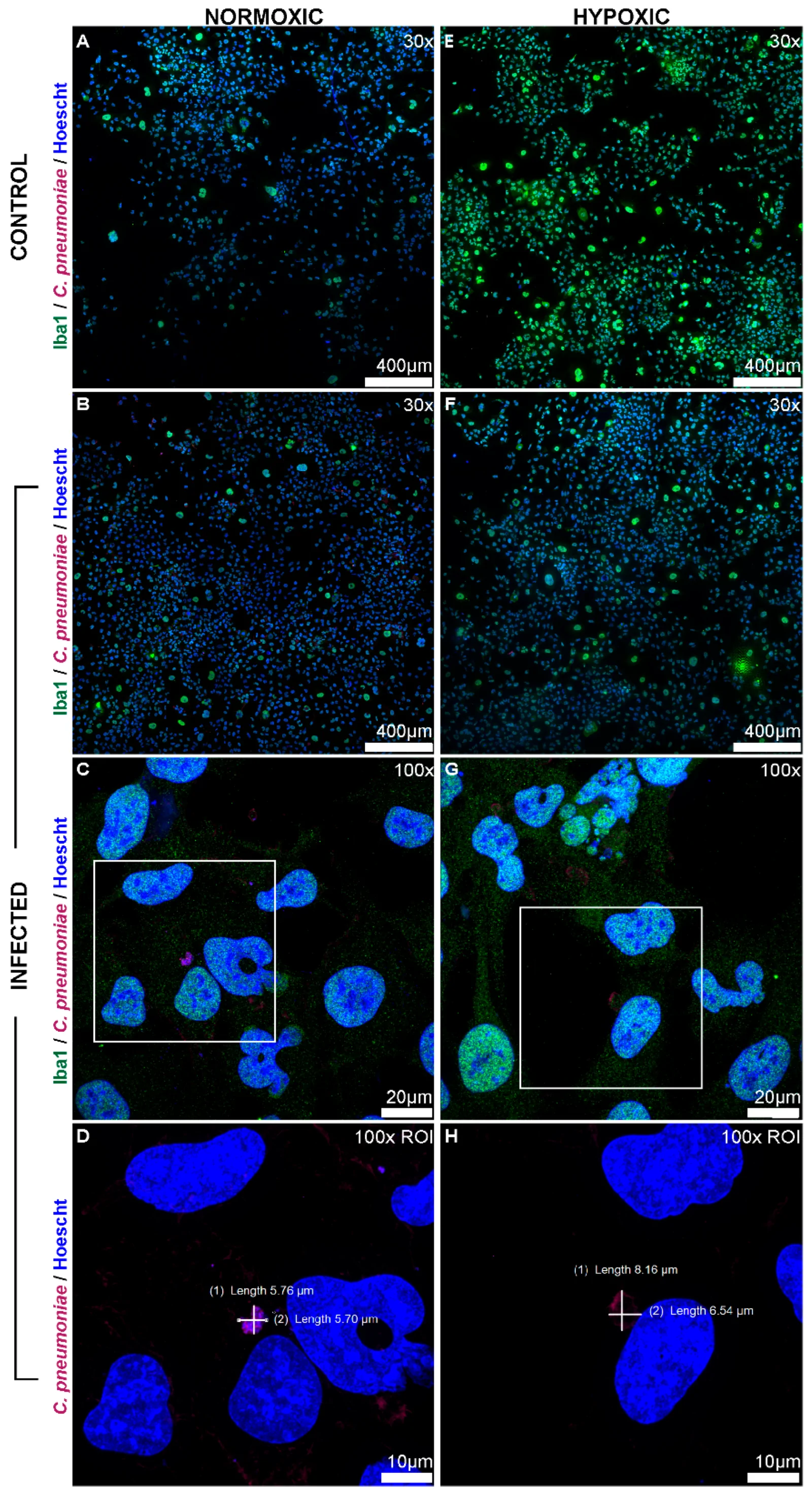
Immunofluorescence confirmation of microglia phenotype and *C. pneumoniae* infection under normoxic and hypoxic conditions. Human HMC3 microglia were maintained under normoxic or hypoxic (3% O_2_) conditions and either left uninfected (control) or infected with *C. pneumoniae* (MOI = 1) for 72 h. (**A**,**E**) Representative 30× images of control microglia under normoxic (**A**) and hypoxic (**E**) conditions. (**B**,**F**) Representative 30× images of *C. pneumoniae*-infected microglia under normoxic (**B**) and hypoxic (**F**) conditions. (**C**,**G**) Representative 100× images of infected microglia under normoxic (**C**) and hypoxic (**G**) conditions, demonstrating intracellular *C. pneumoniae* immunoreactivity. White boxes indicate regions of interest shown as cropped enlargements in (**D**) and (**H**), respectively. (**D**,**H**) Cropped enlargements of the indicated regions from the corresponding 100× images, highlighting intracellular *C. pneumoniae*-positive structures and their measured dimensions. Microglia were labelled for Iba1 (green), *C. pneumoniae* LPS (magenta) and nuclei with Hoechst (blue). Scale bars = 400 µm (**A**,**B**,**E**,**F**), 20 µm (**C**,**G**) and 10 µm (**D**,**H**).

Confocal microscopy images of astrocytes and microglia were obtained after 72 h under four experimental conditions: Normoxia Control, Normoxia Infected, Hypoxia Control and Hypoxia Infected. Hoechst staining identified nuclei (blue). GFAP and Iba1 immunostaining identified astrocytic and microglial phenotypes, respectively, while *C. pneumoniae* LPS immunoreactivity (magenta) was used to assess bacterial localisation. *C. pneumoniae*-positive intracellular structures were observed in infected astrocytes and microglia under both normoxic and hypoxic conditions, including structures localised within the perinuclear region (Figure 1 and Figure 2). Representative 30× images demonstrate the broader cellular distribution, while 100× images and corresponding cropped regions of interest further demonstrate intracellular localisation of *C. pneumoniae*-positive structures.

### 2.2. Infection and Hypoxia Differentially Alter Glial Bioenergetics

**Figure 3:**
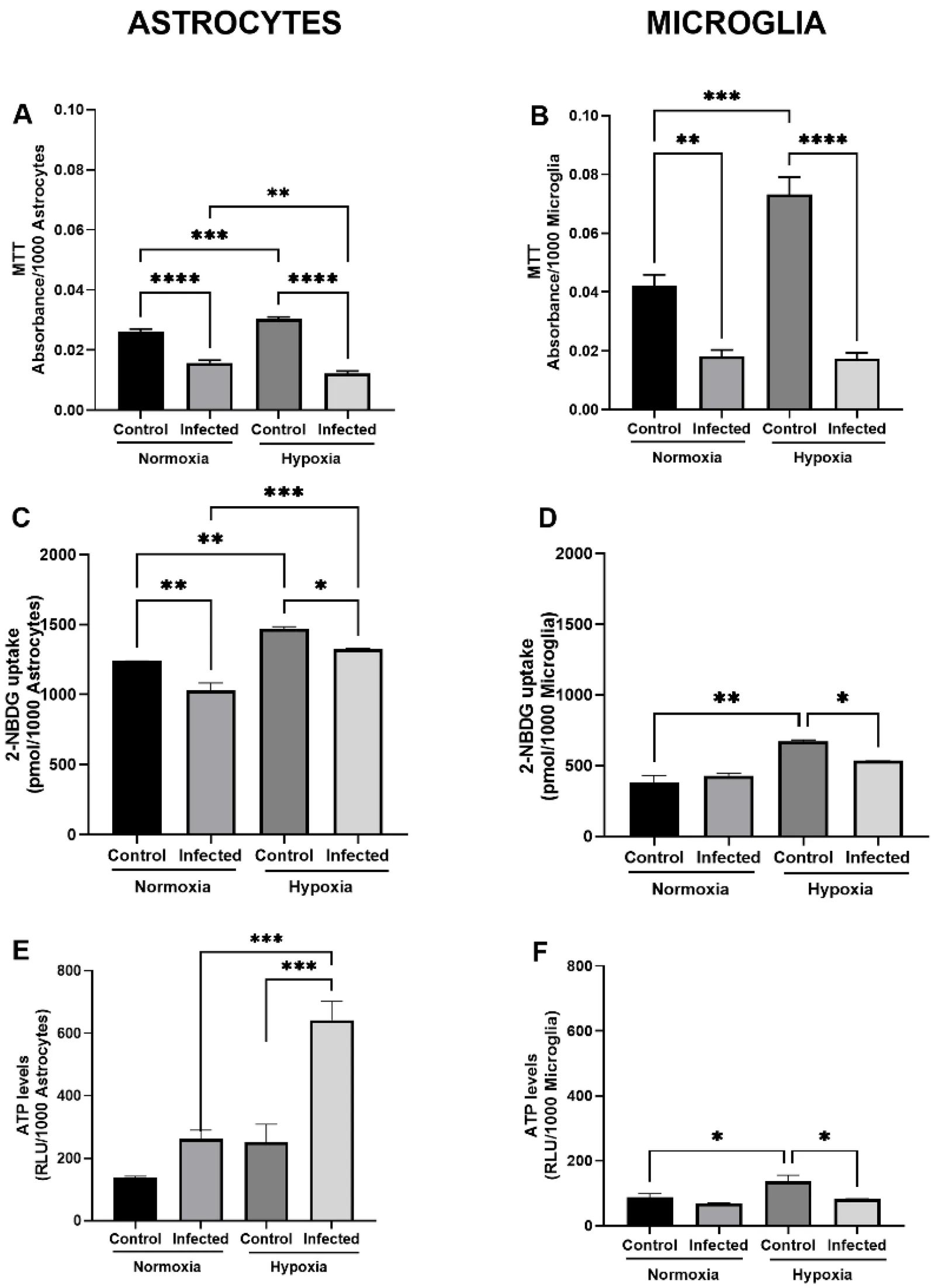
Infection and hypoxia differentially alter glial metabolic responses: astrocytes (**A**,**C**,**E**) and microglia (**B**,**D**,**F**) were infected with either control (PBS) or *C. pneumoniae* (multiplicity of infection (MOI) = 1) and maintained under normoxic or hypoxic (3% O_2_) conditions for 72 h prior to metabolic assessment. (**A**,**B**) MTT reduction assay. (**C**,**D**) Glucose uptake measured using the fluorescent glucose analogue 2-NBDG and normalised to nuclei counts. (**E**,**F**) Intracellular ATP levels measured using a luminescence-based assay and normalised to cell number. Data are presented as mean ± SEM from three independent biological replicates (*n* = 3), with each biological replicate assessed in technical triplicate. Technical replicates were averaged within each biological replicate prior to statistical analysis. Statistical analysis was performed using two-way ANOVA with Tukey’s multiple comparisons test. Adjusted *p*-values were used to determine statistical significance: * *p* < 0.05, ** *p* < 0.01, *** *p* < 0.001, **** *p* < 0.0001.

To determine how infection and oxygen tension influence metabolic state, MTT reduction, glucose uptake and intracellular ATP were assessed 72 h post-infection (Figure 3).

MTT analysis demonstrated that *C. pneumoniae* infection significantly reduced MTT reduction in both astrocytes (Figure 3A) and microglia (Figure 3B) under normoxic conditions compared with non-infected controls. Under hypoxia, MTT activity was significantly increased in both cell types relative to normoxic controls; however, infection significantly reduced MTT reduction compared with hypoxic controls, with the greatest reduction observed in microglia.

Glucose uptake was assessed by 2-NBDG uptake and further distinguished these responses. In astrocytes, infection under normoxia reduced uptake, while hypoxia alone increased uptake relative to the normoxic conditions (Figure 3C). Under combined hypoxia and infection, uptake remained elevated compared with normoxic control but was reduced relative to hypoxia alone. In microglia, hypoxia markedly enhanced uptake; however, infection attenuated this effect under hypoxic conditions (Figure 3D).

Despite reduced MTT reduction, intracellular ATP measurements revealed divergent responses between glial types. In astrocytes, infection under normoxia increased ATP relative to the normoxic control and combined hypoxia and infection significantly increased intracellular ATP levels (Figure 3E). In contrast to astrocytes, microglia displayed reduced ATP under combined hypoxia and infection compared with the hypoxic control (Figure 3F). Hypoxia alone also increased intracellular ATP levels compared with the baseline normoxic control.

### 2.3. Combined Infection and Hypoxia Alter the Extracellular Amyloid-β Peptide Profile

**Figure 4:**
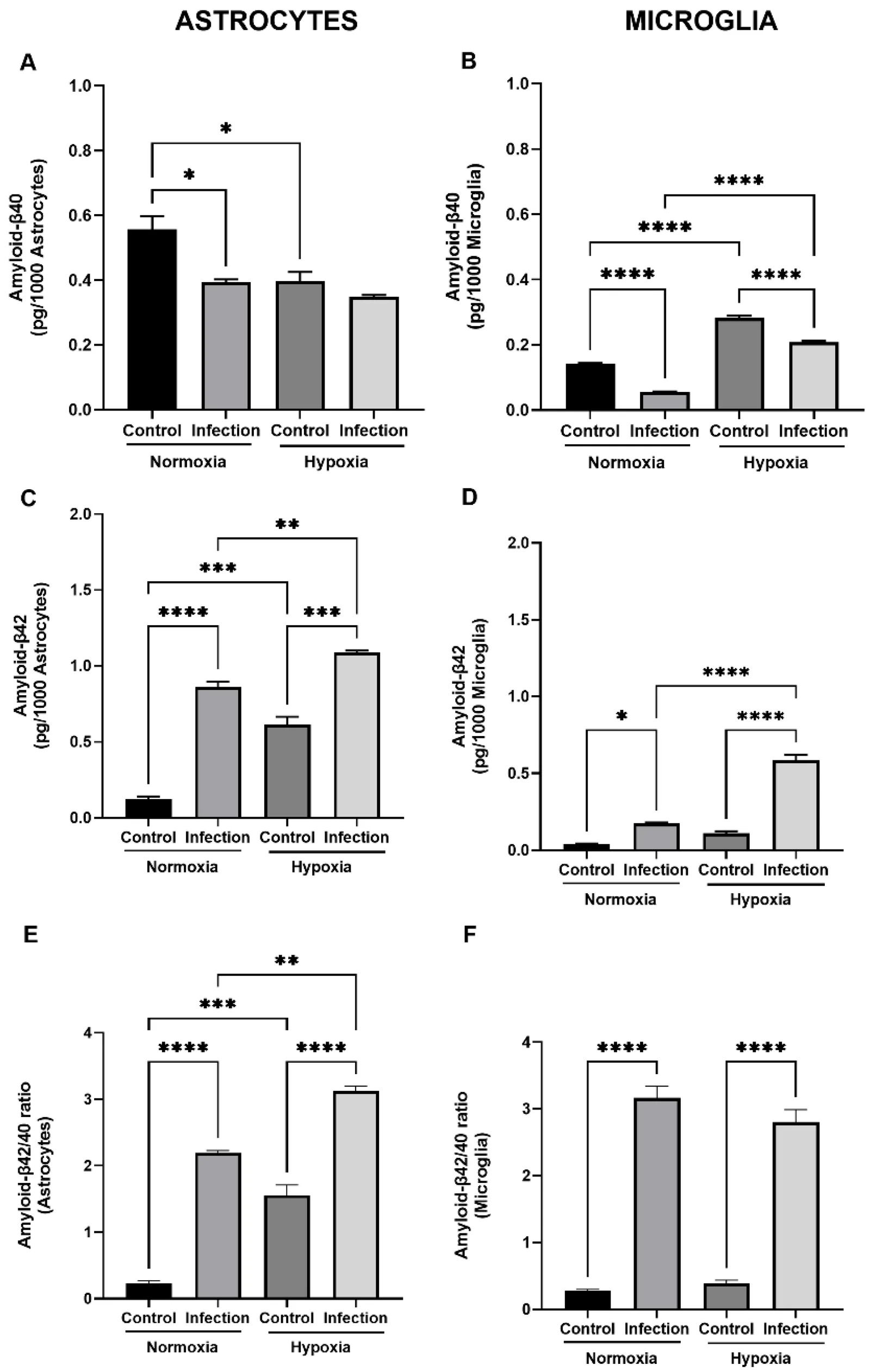
Infection and hypoxia alter the amyloid-β peptide balance in glial cells: astrocytes (**A**,**C**,**E**) and microglia (**B**,**D**,**F**) were infected with *C. pneumoniae* (MOI = 1) and cultured under normoxic or hypoxic (3% O_2_) conditions for 72 h. Aβ40 and Aβ42 levels were measured in culture supernatants using ELISA and normalised to cell number (pg/1000 cells). (**A**,**B**) Secreted Aβ40 levels. (**C**,**D**) Secreted Aβ42 levels. (**E**,**F**) Aβ42/40 ratio calculated from measured peptide concentrations. Data are presented as mean ± SEM from three independent biological replicates (*n* = 3), with each biological replicate assessed in technical triplicate. Technical replicates were averaged within each biological replicate prior to statistical analysis. Statistical analysis was performed using two-way ANOVA with Tukey’s multiple comparisons test. Adjusted *p*-values were used to determine statistical significance * *p* < 0.05, ** *p* < 0.01, *** *p* < 0.001, **** *p* < 0.0001.

To determine whether metabolic remodelling under combined stress was accompanied by alterations in amyloid-β secretion, secreted Aβ40 and Aβ42 concentrations were quantified in culture supernatants 72 h following *C. pneumoniae* infection under normoxic or hypoxic conditions (Figure 4).

In astrocytes, infection significantly reduced Aβ40 secretion under normoxic conditions, whereas hypoxia alone also lowered Aβ40 relative to normoxic controls (Figure 4A). In contrast, Aβ42 secretion increased following infection under both oxygen conditions, with the greatest abundance observed during combined hypoxia and infection (Figure 4C). Consequently, the Aβ42/Aβ40 ratio increased markedly following infection and was further elevated under dual-stress conditions, resulting in an increased relative abundance of the more amyloidogenic Aβ42 species (Figure 4E).

Microglia displayed a distinct response pattern. Aβ40 concentrations varied across conditions, with infection reducing Aβ40 under both normoxic and hypoxic conditions, while hypoxia alone increased Aβ40 relative to normoxic controls (Figure 4B). Aβ42 secretion was also significantly increased by infection, with the largest increase occurring under hypoxic conditions (Figure 4D). As observed in astrocytes, infection significantly increased the Aβ42/Aβ40 ratio under both oxygen conditions, with infected cells exhibiting substantially higher ratios than their respective controls (Figure 4F).

Collectively, these findings demonstrate that *C. pneumoniae* infection alters the extracellular Aβ peptide profile in both astrocytes and microglia, characterised by an increased Aβ42/Aβ40 ratio. In astrocytes, this ratio was further elevated under combined hypoxia and infection, whereas in microglia the increase was predominantly associated with infection and remained elevated under hypoxic conditions. These findings demonstrate treatment-associated alterations in secreted Aβ40 and Aβ42 but do not establish the underlying mechanisms regulating Aβ production or processing.

### 2.4. NanoString Transcriptomic Profiling

**Figure 5:**
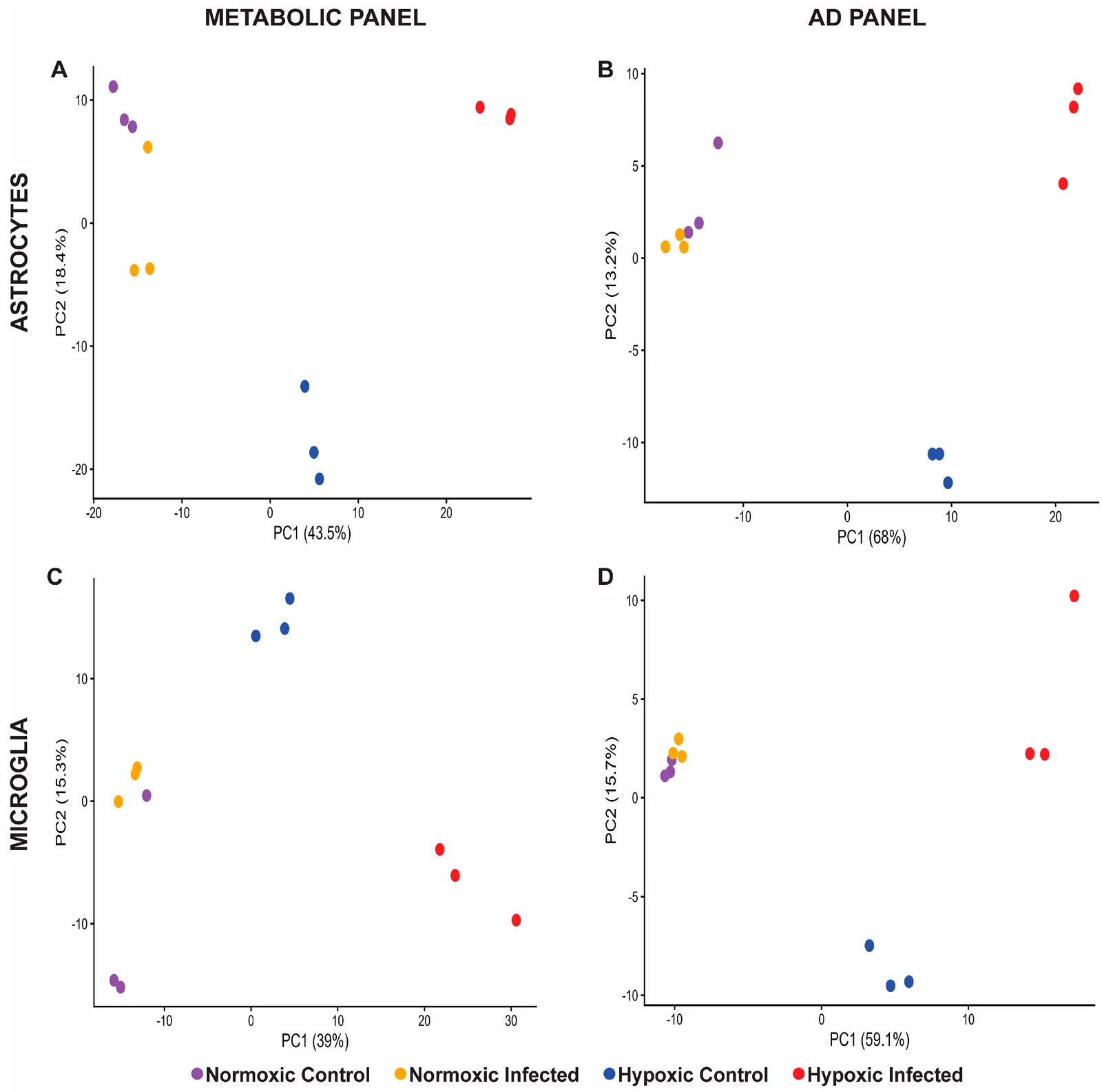
Principal component analysis of NanoString metabolic pathways and Alzheimer’s disease panels in astrocytes and microglia: PCA was performed separately for astrocytes (**A**,**B**) and microglia (**C**,**D**) for the NanoString Metabolic Pathways (**A**–**C**) and Alzheimer’s Disease (**B**–**D**) panels using log_2_-transformed expression values. Samples are coloured by infection status and oxygen conditions (normoxic control, purple circle; normoxic *C. pneumoniae* infection, orange circle; hypoxic control, blue circle; hypoxic *C. pneumoniae* infection, red circle). Separation along the principal components reflects differences in transcriptional profiles associated with cell type, infection status and oxygen tension.

NanoString transcriptomic profiling revealed distinct condition-dependent transcriptional states in astrocytes and microglia. PCA of both the Metabolic Pathways and Alzheimer’s Disease panels showed that oxygen tension was a major driver of transcriptional variation, with clear separation of normoxic and hypoxic samples across the principal components. Infection contributed an additional layer of variation, with separation between infected and control samples particularly apparent under hypoxic conditions. These findings indicate that hypoxia establishes a broad transcriptional state that shapes the response of glial cells to *C. pneumoniae* infection (Figure 5).

#### 2.4.1. Metabolic Panel for Astrocytes

**Figure 6:**
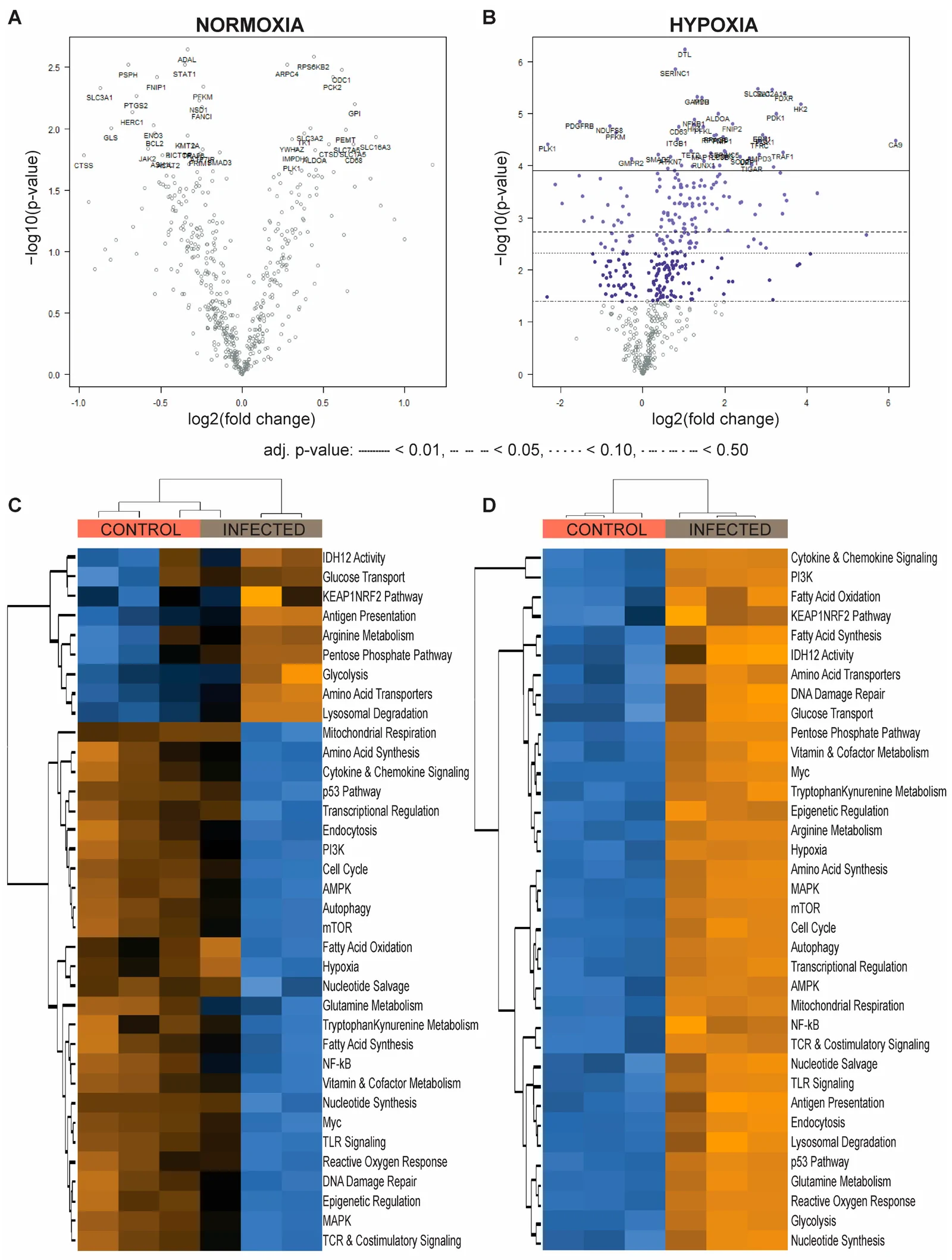
Differential gene expression and pathway scores of the NanoString Metabolic Pathways panel in astrocytes following *C. pneumoniae* infection under normoxic and hypoxic conditions: (**A**,**B**) Volcano plots showing differential gene expression between infected and control astrocytes under normoxia (**A**) and hypoxia (**B**). Each point represents an individual gene plotted according to log_2_ fold change (x-axis) and −log_10_(*p*-value) (y-axis). Horizontal lines indicate adjusted *p*-value (false discovery rate; FDR) thresholds. Purple genes represent genes meeting the selected adjusted *p*-value (FDR) threshold applied in the NanoString nSolver Advanced Analysis workflow, whereas grey genes represent genes that did not meet the selected threshold. (**C**,**D**) Heatmaps showing NanoString pathway scores for control and infected astrocytes under normoxia (**C**) and hypoxia (**D**). Pathway scores are displayed as row-wise Z-scores, with orange indicating relatively higher pathway scores and blue indicating relatively lower pathway scores. Hierarchical clustering was performed on both pathways and samples to identify similarities in pathway score profiles. NanoString analyses were performed using three independent biological replicates per experimental condition (*n* = 3).

Differential gene expression analysis of the NanoString Metabolic Pathways panel demonstrated that the astrocytic transcriptional response to *C. pneumoniae* infection was strongly influenced by oxygen availability. Under normoxic conditions, infection induced only modest transcriptional alterations, with no genes meeting the predefined false discovery rate (FDR) threshold for statistical significance (Figure 6A). Although several genes exhibited altered expression, these changes did not remain significant following multiple testing correction.

In contrast, hypoxia markedly enhanced the transcriptional response to infection (Figure 6B). Numerous genes were significantly differentially expressed between infected and control astrocytes, with the majority demonstrating increased expression in infected cells. Prominent differentially expressed genes included regulators of glucose transport, glycolysis and cellular adaptation to hypoxia, including SLC2A1, SLC2A14, HK2, GAPDH, FDXR, DTL, SERINC1 and MYB (Appendix B.1, Table A2), demonstrating broad transcriptional alterations in metabolic genes under combined hypoxic and infectious stress.

Hierarchical clustering of pathway scores demonstrated distinct pathway profiles between infected and control astrocytes under both oxygen conditions (Figure 6C,D). Under normoxia, pathway-level differences were evident despite the absence of statistically significant differentially expressed genes, suggesting coordinated but relatively subtle alterations in metabolic gene expression following infection. Under hypoxia, however, infected astrocytes exhibited widespread changes in pathway scores relative to controls, with increased scores observed across glycolysis, glucose transport, mitochondrial respiration, amino acid metabolism, pentose phosphate pathway, lysosomal degradation, fatty acid metabolism, cytokine and chemokine signalling, PI3K, mTOR, autophagy, antigen presentation and hypoxia-associated pathways. Collectively, these findings indicate extensive transcriptional regulation of metabolic pathways in astrocytes exposed to combined hypoxic and infectious stress.

Overall, these findings demonstrate that oxygen availability is a major determinant of the astrocytic transcriptional response to *C. pneumoniae*. While infection alone elicited only limited transcriptional changes under normoxic conditions, hypoxia substantially amplified changes in metabolic pathway scores and gene expression, resulting in broad transcriptional alterations associated with cellular metabolism, stress adaptation and inflammatory signalling.

#### 2.4.2. Metabolic Panel for Microglia

**Figure 7:**
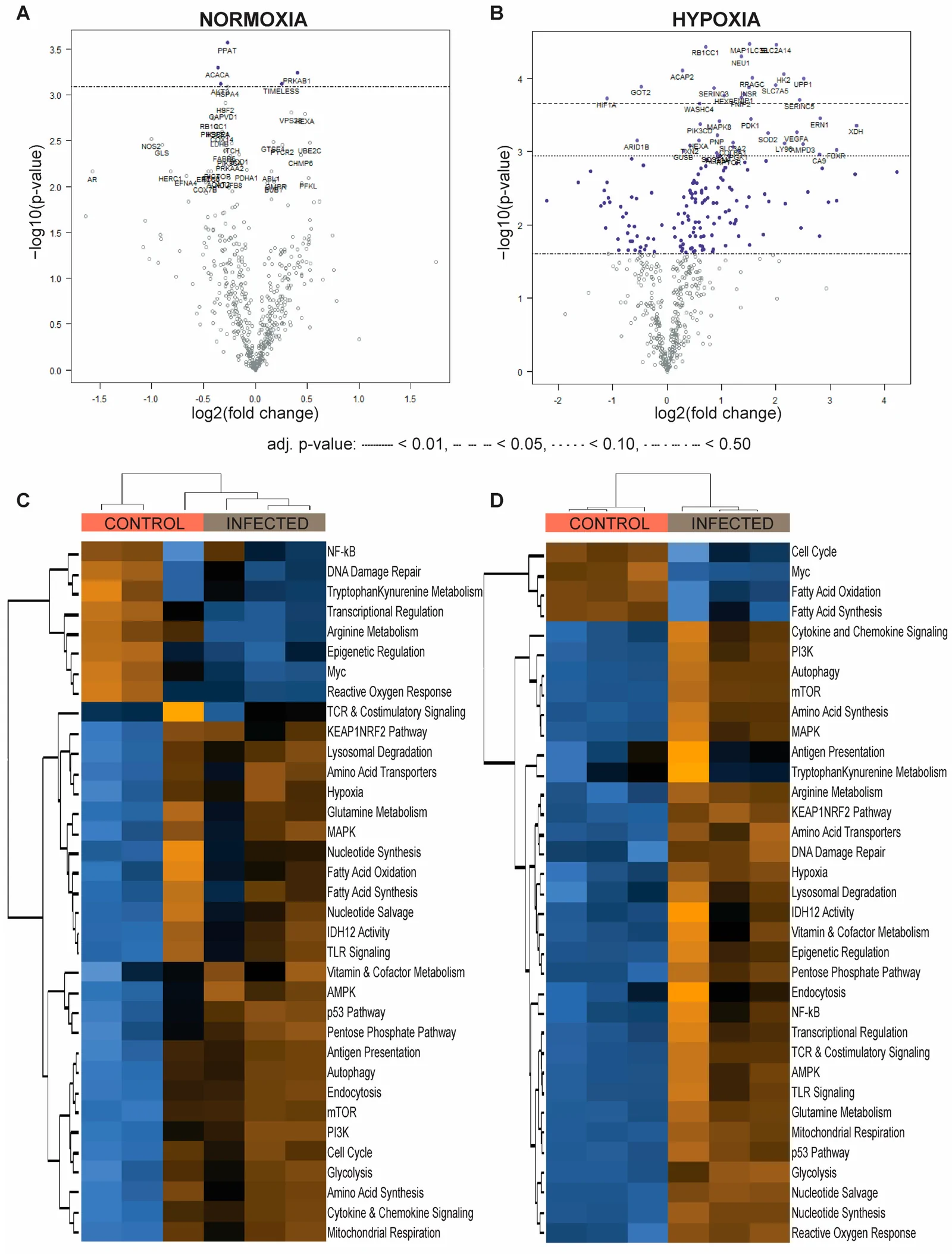
Differential gene expression and pathway scores of the NanoString Metabolic Pathways panel in microglia following *C. pneumoniae* infection under normoxic and hypoxic conditions: (**A**,**B**) Volcano plots showing differential gene expression between infected and control microglia under normoxia (**A**) and hypoxia (**B**). Each point represents an individual gene plotted according to log_2_ fold change (x-axis) and −log_10_(*p*-value) (y-axis). Horizontal lines indicate adjusted *p*-value (false discovery rate; FDR) thresholds. Purple genes represent genes meeting the selected adjusted *p*-value (FDR) threshold applied in the NanoString nSolver Advanced Analysis workflow, whereas grey genes represent genes that did not meet the selected threshold. (**C**,**D**) Heatmaps showing NanoString pathway scores for control and infected microglia under normoxia (**C**) and hypoxia (**D**). Pathway scores are displayed as row-wise Z-scores, with orange indicating relatively higher pathway scores and blue indicating relatively lower pathway scores. Hierarchical clustering was performed on both pathways and samples to identify similarities in pathway score profiles. NanoString analyses were performed using three independent biological replicates per experimental condition (*n* = 3).

Differential gene expression analysis of the NanoString Metabolic Pathways panel revealed that the transcriptional response of microglia to *C. pneumoniae* infection was strongly influenced by oxygen availability. Under normoxic conditions, infection resulted in only modest alterations in gene expression, with no genes meeting the predefined FDR threshold for statistical significance (Figure 7A). Although several genes exhibited trends towards differential expression, these changes did not remain significant following multiple testing corrections.

In contrast, hypoxia markedly enhanced the transcriptional response to infection (Figure 7B). Numerous genes were significantly differentially expressed between infected and control microglia, with the majority demonstrating increased expression in infected cells. Prominent differentially expressed genes included regulators of hypoxic adaptation, glycolytic metabolism, lysosomal function and cellular signalling, including HK2, ACAP2, NEU1, RRAGC, MAP1LC3B and SLC7A5 (Appendix B.1, Table A3), demonstrating broad transcriptional alterations in metabolic genes under combined hypoxic and infectious stress.

Hierarchical clustering of pathway scores demonstrated distinct pathway profiles between infected and control microglia under both oxygen conditions (Figure 7C,D). Under normoxia, pathway-level differences were evident despite the absence of statistically significant differentially expressed genes, suggesting coordinated but relatively subtle transcriptional changes following infection. Under hypoxia, however, infected microglia exhibited widespread changes in scores for metabolic, inflammatory and stress-response pathways relative to controls. Increased pathway scores were observed across glycolysis, mitochondrial respiration, amino acid metabolism, fatty acid metabolism, pentose phosphate pathway, autophagy, mTOR, PI3K, MAPK, cytokine and chemokine signalling, TLR signalling, antigen presentation, reactive oxygen species signalling and hypoxia-associated pathways, indicating extensive transcriptional regulation of metabolic pathways during combined hypoxic and infectious stress (Figure 7D).

Collectively, these findings demonstrate that oxygen availability is a major determinant of the microglial transcriptional response to *C. pneumoniae*. While infection alone elicited only limited transcriptional changes under normoxic conditions, hypoxia substantially amplified metabolic and inflammatory gene expression, resulting in broad transcriptional alterations in pathways associated with cellular metabolism, stress adaptation and innate immune function.

#### 2.4.3. Differential Effects of Hypoxia on Astrocytes and Microglia

**Figure 8:**
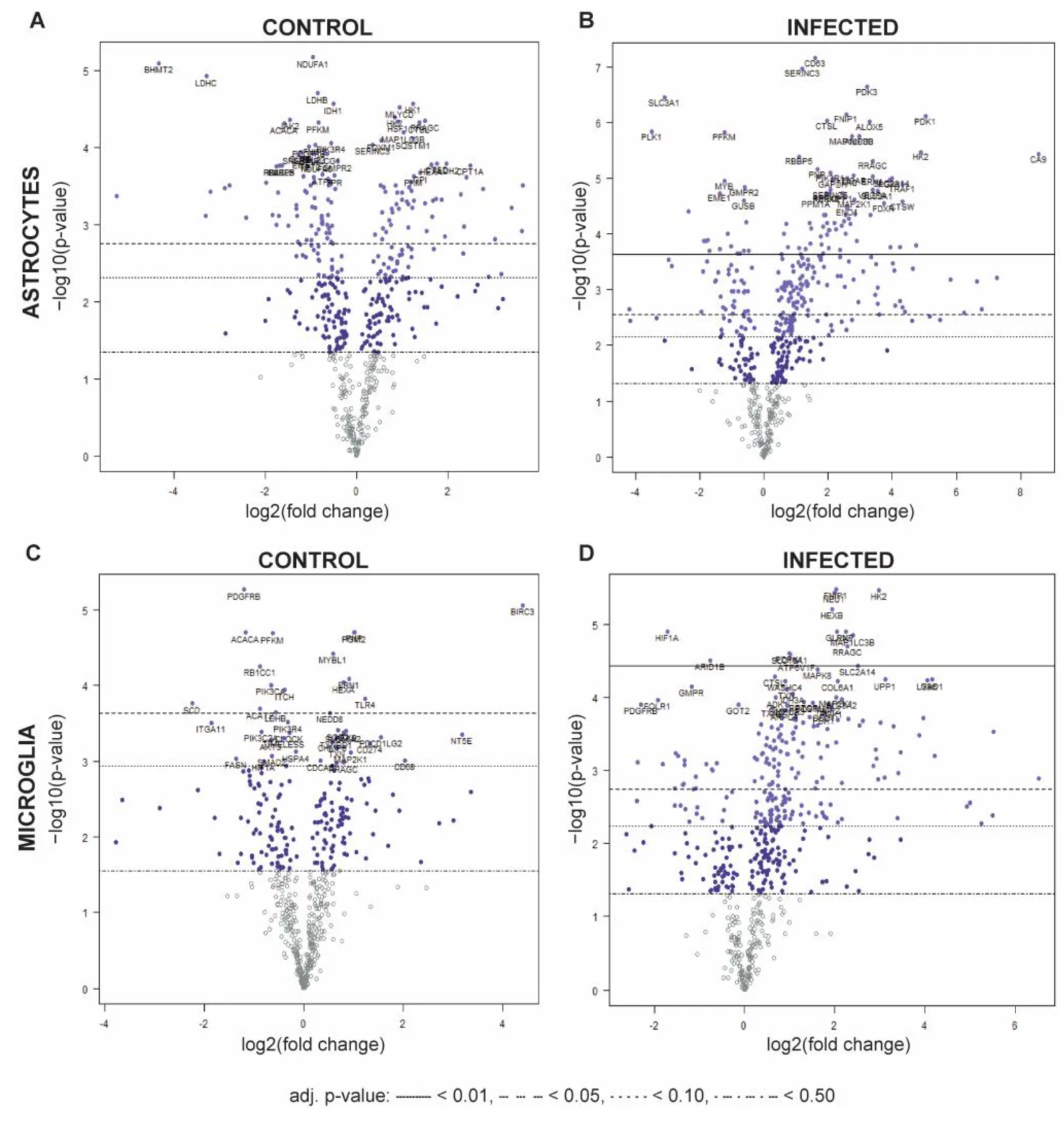
Differential metabolic gene expression associated with hypoxia in astrocytes and microglia. (**A**,**B**) Volcano plots showing differential gene expression between hypoxic and normoxic astrocytes under control (**A**) and *C. pneumoniae*-infected (**B**) conditions. (**C**,**D**) Volcano plots showing differential gene expression between hypoxic and normoxic microglia under control (**C**) and *C. pneumoniae*-infected (**D**) conditions. Each point represents an individual gene plotted according to log_2_ fold change (hypoxia–normoxia; x-axis) and −log_10_(*p*-value) (y-axis). Horizontal lines indicate adjusted *p*-value (false discovery rate; FDR) thresholds. Purple genes represent genes meeting the selected adjusted *p*-value (FDR) threshold applied in the NanoString nSolver Advanced Analysis workflow, whereas grey genes represent genes that did not meet the selected threshold. NanoString analyses were performed using three independent biological replicates per experimental condition (*n* = 3).

To determine the effect of oxygen availability on metabolic gene expression, differential expression analysis was performed by comparing hypoxic and normoxic astrocytes and microglia under both control and *C. pneumoniae*-infected conditions (Figure 8). Across both glial cell types, hypoxia induced widespread transcriptional remodelling relative to normoxia, demonstrating that oxygen availability was the primary driver of metabolic gene expression. The top significantly differentially expressed genes are presented in Appendix B.1, Table A4, Table A5, Table A6 and Table A7.

In astrocytes, hypoxia induced extensive differential gene expression under both control (Figure 8A) and infected (Figure 8B) conditions. In control astrocytes, hypoxia significantly altered the expression of genes involved in glycolysis, mitochondrial metabolism and nutrient sensing, including HK1, HK2, NDUFA1, IDH1, MLYCD and RRAGC (Appendix B.1, Table A4). In infected astrocytes, hypoxia was associated with a broader transcriptional response characterised by significant changes in PDK1, PDK3, CD63, SERINC3, CTSL, FNIP1, ALOX5, PLK1, PFKM and SLC3A1 (Appendix B.1, Table A5), consistent with enhanced metabolic adaptation and cellular stress responses during infection.

Microglia exhibited a similar but distinct transcriptional response to hypoxia. In control microglia (Figure 8C), hypoxia significantly altered genes involved in energy metabolism, cell signalling and lysosomal function, including PDGFRB, BIRC3, PNP, PGM2, ACACA, PFKM, MYBL1, RB1CC1, ERN1 and HEXA (Appendix B.1, Table A6). Under infected conditions (Figure 8D), hypoxia elicited a more extensive transcriptional response, with significant differential expression of FNIP1, HK2, NEU1, HEXB, PNP, HIF1A, GLRX, MAP1LC3B, RRAGC and PDPK1 (Appendix B.1, Table A7), indicating activation of pathways associated with glycolytic metabolism, lysosomal function, autophagy and hypoxic adaptation.

Collectively, these findings demonstrate that hypoxia is the dominant driver of metabolic transcriptional remodelling in both astrocytes and microglia. While substantial hypoxia-induced gene expression changes occurred in both control and infected cells, infection altered the transcriptional profile by engaging additional genes involved in metabolic adaptation, lysosomal function and cellular stress responses.

#### 2.4.4. Comparison of Alzheimer’s Disease-Associated Gene Expression Between Astrocytes and Microglia

**Figure 9:**
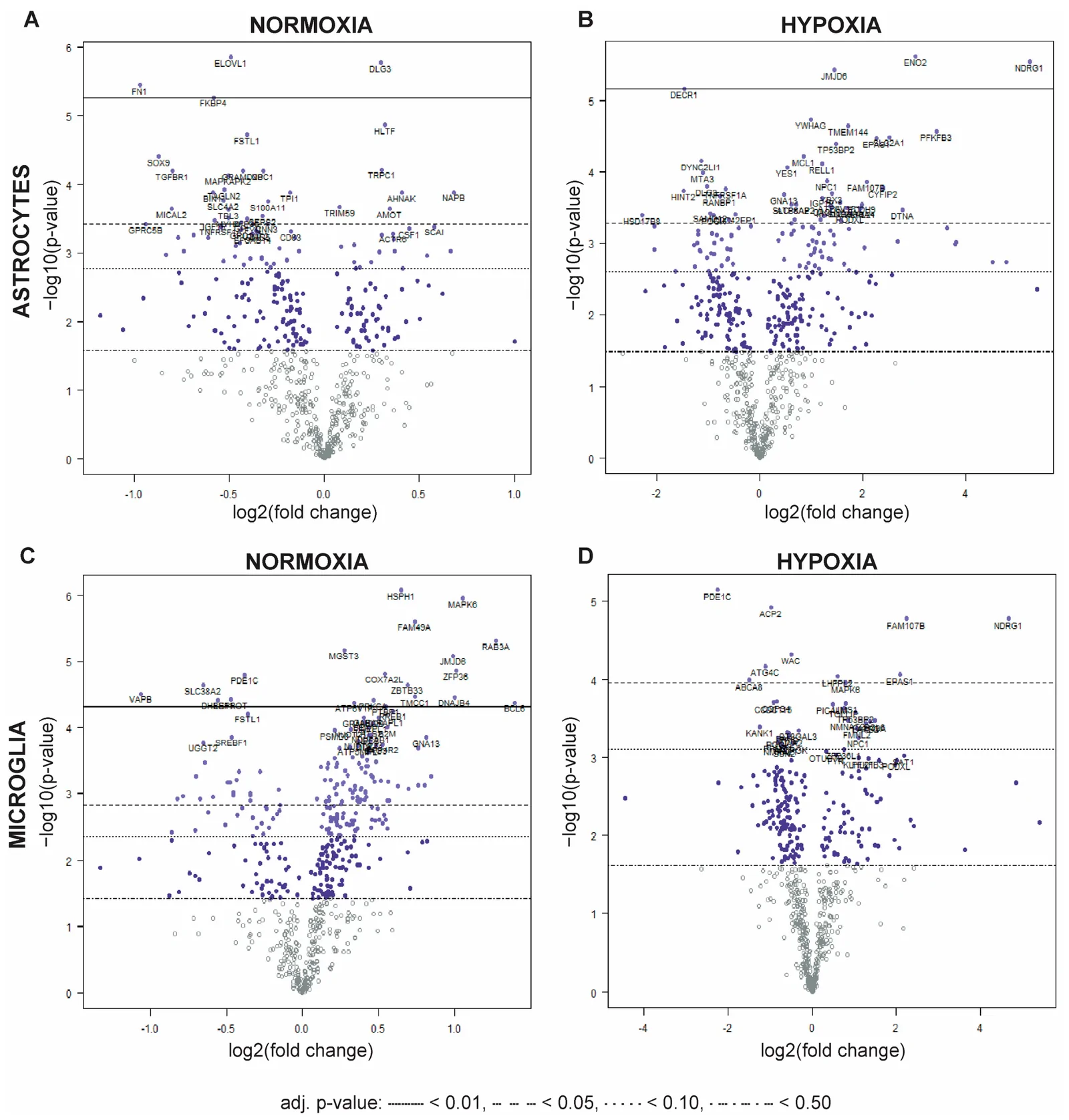
Differential gene expression of the NanoString Alzheimer’s Disease panel following *C. pneumoniae* infection under normoxic and hypoxic conditions. Volcano plots showing differential gene expression between infected and control astrocytes under normoxia (**A**) and hypoxia (**B**), and infected and control microglia under normoxia (**C**) and hypoxia (**D**). Each point represents an individual gene plotted according to log_2_ fold change (x-axis) and −log_10_(*p*-value) (y-axis). Horizontal lines indicate adjusted *p*-value (false discovery rate; FDR) thresholds. Purple genes represent genes meeting the selected adjusted *p*-value (FDR) threshold applied in the NanoString nSolver Advanced Analysis workflow, whereas grey genes represent genes that did not meet the selected threshold. NanoString analyses were performed using three independent biological replicates per experimental condition (*n* = 3).

Differential expression analysis of the NanoString Alzheimer’s Disease panel demonstrated distinct transcriptional responses to *C. pneumoniae* infection in astrocytes and microglia under both normoxic and hypoxic conditions (Figure 9). The pattern of differentially expressed genes varied according to both cell type and oxygen availability, indicating that the transcriptional response of Alzheimer’s disease-associated pathways is influenced by the combined effects of infection and hypoxia. The top significantly differentially expressed genes are presented in Appendix B.2, Table A8, Table A9, Table A10 and Table A11.

Under normoxic conditions, infected astrocytes exhibited significant differential expression of genes involved in lipid metabolism, extracellular matrix organisation and cellular signalling, including ELOVL1, FN1, FKBP4, FSTL1, SOX9, TGFBR1 and NPC1, together with increased expression of DLG3, HLTF and TRPC1 (Figure 9A). Under hypoxia, the astrocytic response shifted towards genes associated with hypoxic adaptation, glucose metabolism and cellular stress, including ENO2, NDRG1, JMJD6, PFKFB3, SLC2A1, EPAS1, TMEM144, TP53BP2, DECR1 and YWHAG (Figure 9B).

Microglia displayed a distinct transcriptional profile compared with astrocytes. Under normoxic conditions, infection significantly altered the expression of HSPH1, MAPK6, FAM49A, RAB3A, MGST3, JMJD6, ZFP36, COX7A2L, PDE1C and ZBTB33 (Figure 9C). Under hypoxic conditions, the transcriptional response was characterised by significant changes in PDE1C, ACP2, FAM107B, NDRG1, WAC, ATG4C, EPAS1, LHFPL2, ABCA8 and MAPK6 (Figure 9D), demonstrating a distinct pattern of Alzheimer’s disease-associated gene expression under combined hypoxic and infectious stress.

Collectively, these findings demonstrate that both oxygen availability and cell type shape the Alzheimer’s disease-associated transcriptional response to *C. pneumoniae* infection. Whereas astrocytes exhibited prominent alterations in genes associated with lipid metabolism, hypoxic adaptation and glucose metabolism, microglia displayed differential regulation of genes linked to cellular signalling, stress responses and lysosomal-associated processes, highlighting distinct glial responses to infection under normoxic and hypoxic conditions.

#### 2.4.5. Factorial Analysis of Infection × Oxygen Interaction Effects

**Figure 10:**
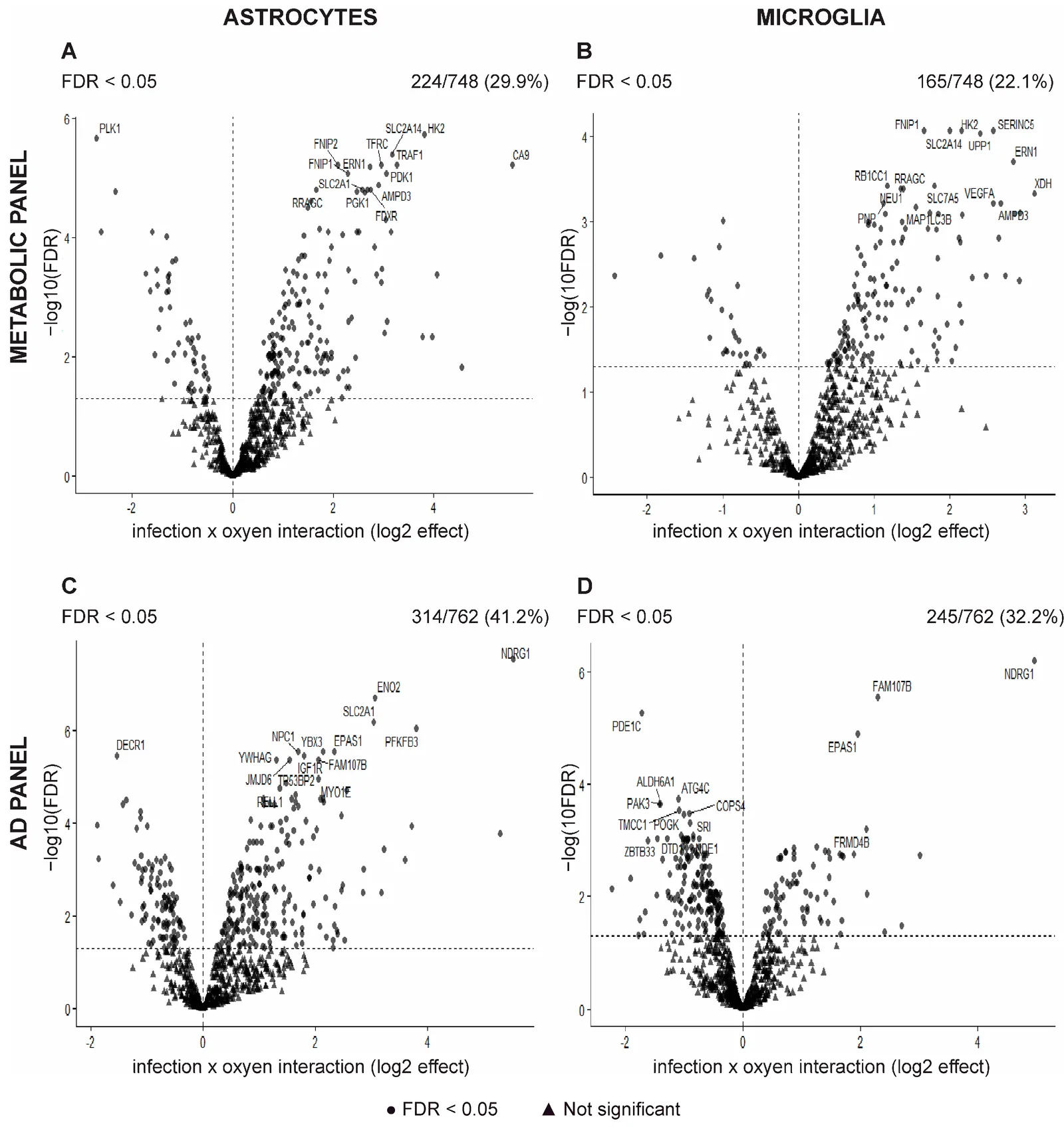
Factorial analysis of infection × oxygen interaction effects across NanoString Metabolic Pathways and Alzheimer’s Disease panels in astrocytes and microglia. (**A**,**B**) Volcano plots showing infection × oxygen interaction effects for genes within the Metabolic Pathways panel in astrocytes (**A**) and microglia (**B**). (**C**,**D**) Volcano plots showing infection × oxygen interaction effects for genes within the Alzheimer’s Disease (AD) panel in astrocytes (**C**) and microglia (**D**). The x-axis represents the infection × oxygen interaction effect (log_2_ effect), corresponding to the difference in the effect of *C. pneumoniae* infection between hypoxic and normoxic conditions and the y-axis represents −log_10_(FDR). Circles represent genes with significant interaction effects (FDR < 0.05), whereas triangles represent genes that did not meet the FDR threshold. Vertical dashed lines indicate interaction effect sizes of ±1 log_2_ and the horizontal dashed line indicates the FDR < 0.05 threshold. The number and percentage of genes exhibiting significant interaction effects are indicated for each panel. Factorial analyses were performed using three independent biological replicates per experimental condition (*n* = 3).

To determine whether the transcriptional response to *C. pneumoniae* infection was dependent on oxygen availability, a factorial analysis was performed incorporating oxygen condition, infection status and the oxygen × infection interaction (Figure 10). The interaction term represents the difference in the effect of infection between hypoxic and normoxic conditions, thereby identifying genes for which the transcriptional response to infection differed according to oxygen availability.

Significant oxygen × infection interactions were identified across both NanoString panels and in both glial cell types. Within the Metabolic Pathways panel, significant interaction effects were observed for 224 of 748 genes (29.9%) in astrocytes and 165 of 748 genes (22.1%) in microglia (FDR < 0.05; Figure 10A,B). In astrocytes, prominent interaction effects included *HK2*, *SLC2A14*, *TFRC*, *CA9* and *PLK1*, while microglial interaction effects included *HK2*, *SLC2A14*, *FNIP1*, *SERINC5*, *UPP1* and *RRAGC*. These findings demonstrate that the transcriptional effects of infection on metabolic genes differed substantially between normoxic and hypoxic conditions. The top significantly differentially expressed genes are presented in Supplementary Tables S6–S9.

The Alzheimer’s Disease panel demonstrated an even greater proportion of significant interaction effects, with 314 of 762 genes (41.2%) in astrocytes and 245 of 762 genes (32.2%) in microglia meeting the FDR < 0.05 threshold (Figure 10C,D). Prominent interaction effects in astrocytes included *NDRG1*, *ENO2*, *SLC2A1*, *PFKFB3*, *EPAS1* and *NPC1*, while *NDRG1*, *FAM107B* and *EPAS1* were among the prominent positive interaction effects in microglia.

Collectively, the factorial analysis demonstrates that oxygen availability significantly modified the transcriptional response to *C. pneumoniae* infection across both metabolic and AD-related gene panels. The greater proportion of significant interaction effects observed in the AD panel, particularly in astrocytes, further indicates that the combined influence of infection and oxygen availability extends beyond metabolic genes to broader AD-related molecular pathways.

### 2.5. Proteomic Profiling Reveals Oxygen-Dependent Modulation of Infection-Associated Protein Networks in Glial Cells

To complement the transcriptomic analyses, quantitative Tandem Mass Tag (TMT)-based proteomic profiling was performed to investigate infection-associated changes in protein abundance in astrocytes and microglia cultured under normoxic and hypoxic conditions. Differential protein abundance was assessed by comparing infected and control cells separately under each oxygen condition, followed by canonical pathway enrichment analysis of significantly altered proteins. PCA was used to examine global proteomic variation across experimental groups. Together, these analyses were used to determine how oxygen tension influences the proteomic response of glial cells to *C. pneumoniae* infection.

#### 2.5.1. Astrocytic Proteomic Response to *C. pneumoniae* Infection Under Normoxic and Hypoxic Conditions

**Figure 11:**
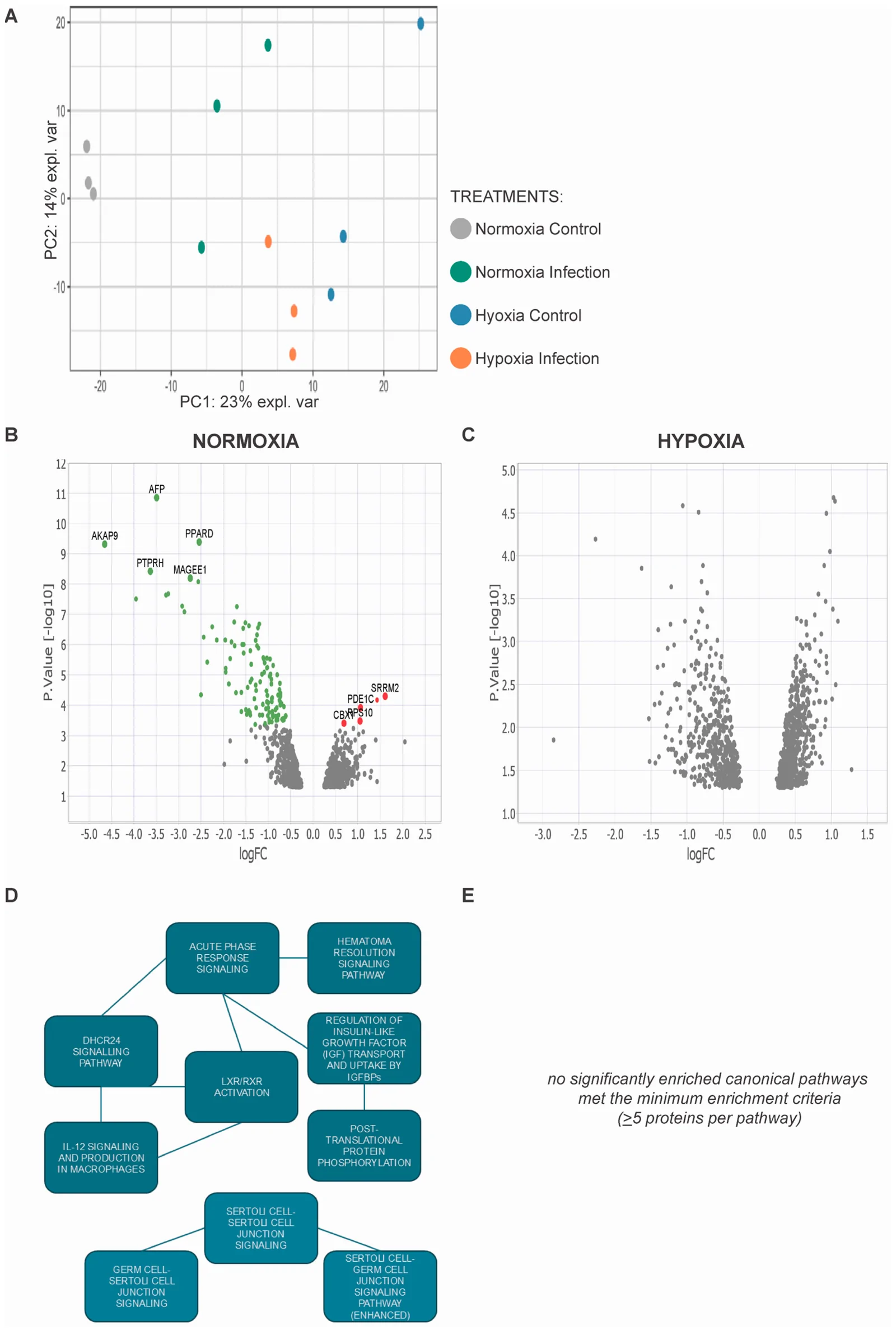
Astrocytic proteomic response to *C. pneumoniae* infection under normoxic and hypoxic conditions. (**A**) PCA of TMT-based proteomic profiles showing global clustering of astrocyte samples according to oxygen condition and infection status. (**B**) Volcano plot showing differentially abundant proteins between infected and control astrocytes under normoxic conditions. (**C**) Volcano plot showing differentially abundant proteins between infected and control astrocytes under hypoxic conditions. Proteins are plotted according to log_2_ fold change (x-axis) and −log_10_(*p*-value) (y-axis), with green and red proteins representing significantly decreased and increased protein abundance, respectively (FDR < 0.05 and |log_2_ fold change| > 0.58). (**D**) Canonical pathway enrichment analysis of significantly differentially abundant proteins identified under normoxic conditions. (**E**) Canonical pathway enrichment analysis of significantly differentially abundant proteins identified under hypoxic conditions. No canonical pathways met the minimum enrichment criteria (≥5 proteins per pathway) under hypoxic conditions. Proteomic analyses were performed using three independent biological replicates per condition (*n* = 3).

Proteomic profiling was performed to investigate the effects of *C. pneumoniae* infection on astrocytic protein abundance under normoxic and hypoxic conditions (Figure 11).

PCA demonstrated clear separation of samples according to oxygen tension, with normoxic and hypoxic astrocytes forming distinct clusters. Within each oxygen condition, infected samples also separated from their respective controls, indicating that both oxygen availability and infection contributed to variation in the astrocytic proteome (Figure 11A).

Under normoxic conditions, infection induced widespread alterations in protein abundance, with numerous significantly differentially abundant proteins identified between infected and control astrocytes (Figure 11B). Canonical pathway enrichment analysis demonstrated significant enrichment of multiple biological pathways, including; Acute Phase Response Signalling, LXR/RXR Activation, IL-12 Signalling and Production in Macrophages, DHCR24 Signalling, Regulation of Insulin-like Growth Factor (IGF) Transport and Uptake by IGFBPs, Hematoma Resolution Signalling, Post-Translational Protein Phosphorylation and Sertoli Cell–Sertoli Cell Junction Signalling (Figure 11D), indicating broad proteomic remodelling following infection under normoxic conditions.

In contrast, infection under hypoxic conditions produced comparatively limited changes in protein abundance (Figure 11C). Although PCA demonstrated clear separation between hypoxic infected and control samples (Figure 11A), no proteins met the significance threshold for differential abundance and no canonical pathways satisfied the minimum enrichment criterion of at least five proteins per pathway (Figure 11E).

Collectively, these findings demonstrate that oxygen availability substantially influenced the astrocytic proteomic response to *C. pneumoniae* infection. Whereas infection under normoxia resulted in widespread alterations in protein abundance and pathway enrichment, these proteomic changes were markedly attenuated under hypoxic conditions despite distinct clustering of experimental groups.

#### 2.5.2. Microglial Proteomic Response to *C. pneumoniae* Infection Under Normoxic and Hypoxic Conditions

**Figure 12:**
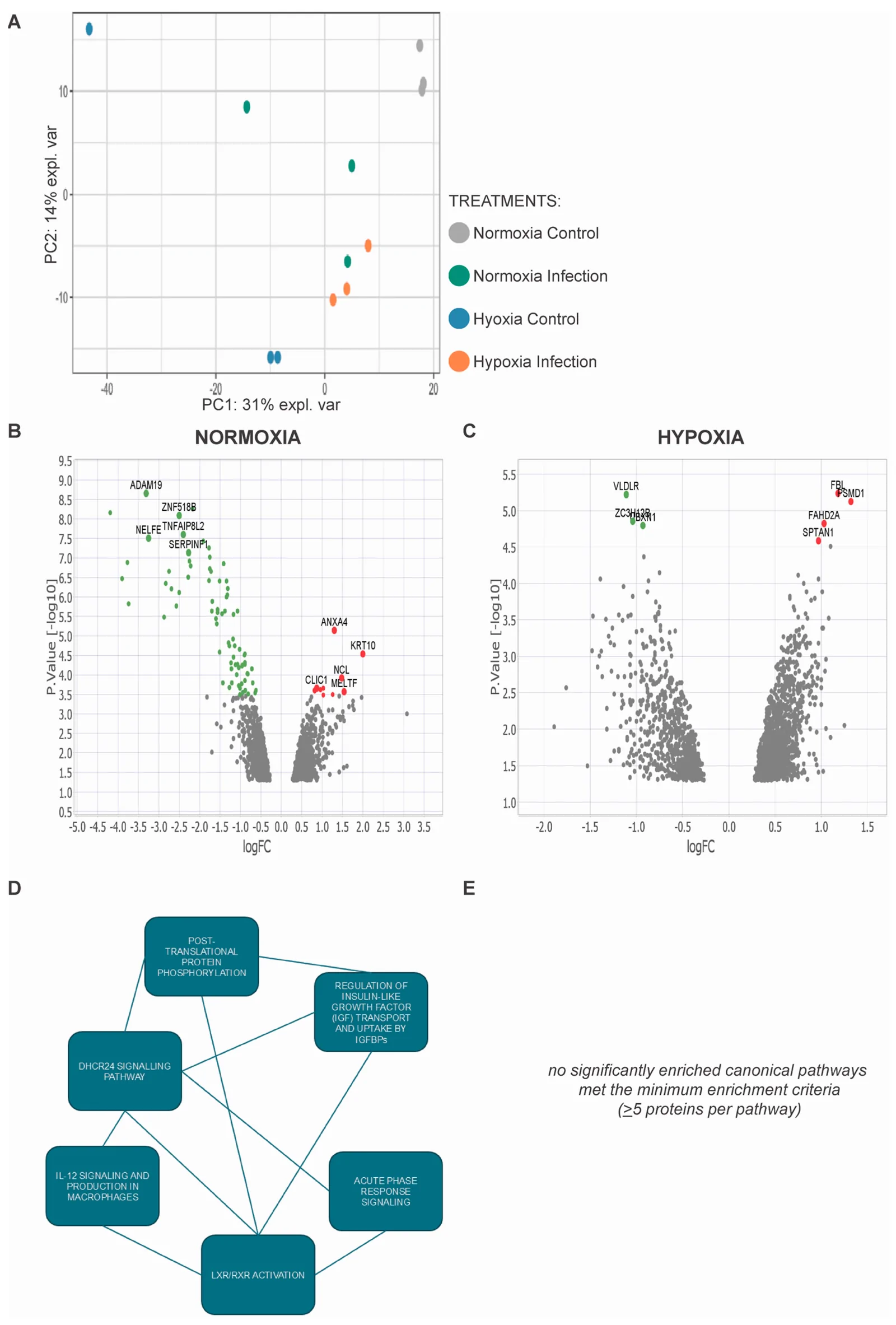
Microglial proteomic response to *C. pneumoniae* infection under normoxic and hypoxic conditions. (**A**) PCA of TMT-based proteomic profiles showing global clustering of microglial samples according to oxygen condition and infection status. (**B**) Volcano plot showing differentially abundant proteins between infected and control microglia under normoxic conditions. (**C**) Volcano plot showing differentially abundant proteins between infected and control microglia under hypoxic conditions. Proteins are plotted according to log_2_ fold change (x-axis) and −log_10_(*p*-value) (y-axis), with green and red proteins representing significantly decreased and increased protein abundance, respectively (FDR < 0.05 and |log_2_ fold change| > 0.58). (**D**) Canonical pathway enrichment analysis of significantly differentially abundant proteins identified under normoxic conditions. (**E**) Canonical pathway enrichment analysis of significantly differentially abundant proteins identified under hypoxic conditions. No canonical pathways met the minimum enrichment criteria (≥5 proteins per pathway). Proteomic analyses were performed using three independent biological replicates per condition (*n* = 3).

Proteomic profiling was performed to investigate the effects of *C. pneumoniae* infection on microglial protein abundance under normoxic and hypoxic conditions (Figure 12). 

PCA demonstrated clear separation of samples according to oxygen tension, with normoxic and hypoxic microglia forming distinct clusters. Within each oxygen condition, infected samples also separated from their respective controls, indicating that both oxygen availability and infection contributed to variation in the microglial proteome (Figure 12A).

Under normoxic conditions, infection induced widespread alterations in protein abundance, with numerous significantly differentially abundant proteins identified between infected and control microglia (Figure 12B). Canonical pathway enrichment analysis identified significant enrichment of LXR/RXR Activation, Acute Phase Response Signalling, IL-12 Signalling and Production in Macrophages, DHCR24 Signalling, Post-Translational Protein Phosphorylation and Regulation of Insulin-like Growth Factor (IGF) Transport and Uptake by IGF Binding Proteins (IGFBPs) (Figure 12D), indicating substantial proteomic remodelling following infection under normoxic conditions.

Under hypoxic conditions, differential protein abundance analysis also identified significantly altered proteins between infected and control microglia (Figure 12C). However, despite these changes and the separation of experimental groups observed by PCA (Figure 12A), no canonical pathways satisfied the minimum enrichment criterion of at least five proteins per pathway (Figure 12E).

Collectively, these findings demonstrate that oxygen availability influenced the microglial proteomic response to *C. pneumoniae* infection. While infection altered protein abundance under both normoxic and hypoxic conditions, coordinated pathway-level enrichment was observed only under normoxia, suggesting that hypoxia attenuated broader functional proteomic responses despite the presence of significantly differentially abundant proteins.

#### 2.5.3. Factorial Analysis of Infection × Oxygen Interaction Effects on Glial Proteomic Responses 

**Figure 13:**
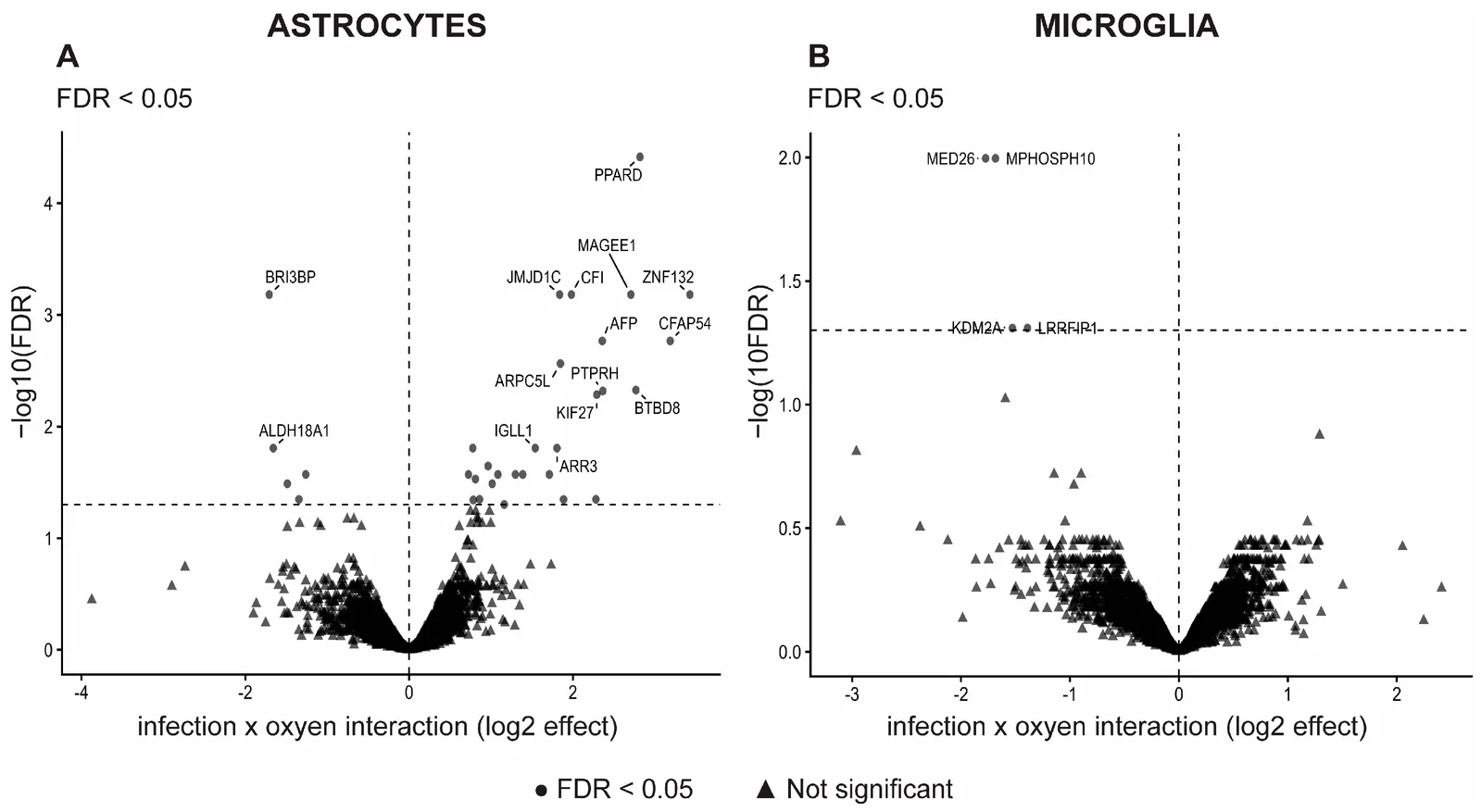
Oxygen-dependent effects of *C. pneumoniae* infection on protein abundance in astrocytes and microglia. Volcano plots show oxygen × infection interaction effects in (**A**) astrocytes and (**B**) microglia following factorial analysis of normalised protein-level abundances. The x-axis represents the infection × oxygen interaction effect on the log2 scale, calculated as (hypoxic infected − hypoxic control) − (normoxic infected − normoxic control), and the y-axis represents −log10(FDR). Proteins with significant interaction effects were defined using a Benjamini–Hochberg-adjusted FDR < 0.05. Selected significant proteins are labelled by human gene symbol. Positive interaction effects indicate a more positive infection-associated change under hypoxia relative to normoxia, whereas negative interaction effects indicate a more negative infection-associated change under hypoxia relative to normoxia.

Factorial analysis was additionally performed to determine whether infection-associated changes in protein abundance differed according to oxygen availability. Significant oxygen × infection interaction effects were identified for 32 of 5338 proteins (0.6%) in astrocytes and 4 of 5062 proteins (0.08%) in microglia (Figure 13).

In astrocytes, the strongest interaction effect was observed for PPARD, with additional significant interactions including CFI, MAGEE1, BRI3BP, and ZNF132. In microglia, significant interaction effects were identified for MPHOSPH10, MED26, LRRFIP1, and KDM2A. These findings indicate that oxygen availability modified the infection-associated protein abundance response for a relatively restricted subset of proteins, particularly in astrocytes.

## 3. Discussion

This study provides a comprehensive multi-omics investigation of how hypoxia influences glial responses to *C. pneumoniae* infection. By integrating functional metabolic assays with transcriptomic and proteomic profiling, we demonstrate that oxygen availability is a major determinant of glial responses to intracellular infection. Although hypoxia profoundly remodelled transcriptional programmes in both astrocytes and microglia, its effects on protein abundance were markedly different, highlighting complex regulation across multiple molecular levels. Factorial proteomic analysis further demonstrated significant oxygen × infection interactions in a restricted subset of proteins, indicating that oxygen availability also modifies specific infection-associated responses at the protein level. Together, these findings demonstrate that *C. pneumoniae* infection and hypoxia interact to reshape glial function in a manner that is highly dependent on cellular lineage and environmental context. Furthermore, they support the concept that microbial infection and hypoxia may converge to influence AD-relevant changes in glial metabolism and Aβ homeostasis [70,71,72,73].

Functional metabolic analyses revealed noticeable differences in the capacity of astrocytes and microglia to adapt to combined stress. Although MTT reduction was reduced following infection and combined stress, astrocytes exhibited significantly increased intracellular ATP despite reduced MTT reduction, indicating divergent changes in cellular reductive activity and intracellular energy status under infectious and hypoxic stress. In contrast, microglia exhibited reduced ATP levels under combined stress, consistent with reduced energetic capacity under these conditions. These findings are consistent with the established role of astrocytes as metabolic support cells capable of adapting to environmental stress [50,74], whereas microglia displayed greater disruption of ATP homeostasis under combined stress [69,75,76]. Glial metabolic dysfunction is increasingly recognized as a central feature of AD and has been linked to impaired neuroprotection, chronic neuroinflammation and disease progression.

Glucose uptake measurements further highlighted lineage-specific responses. Hypoxia increased glucose uptake in both glial populations, consistent with increased cellular glucose uptake under low-oxygen conditions [77]. However, infection attenuated this response in microglia under hypoxic conditions, indicating that infection modifies the glucose uptake response to hypoxia. In contrast, astrocytes maintained relatively elevated glucose uptake under combined stress, demonstrating distinct regulation of glucose uptake between the two glial cell types. Such differences in metabolic adaptability may have important consequences for glial function in the ageing brain, where chronic hypoperfusion and reduced oxygen availability are common pathological features.

NanoString profiling revealed that oxygen availability was the principal determinant of transcriptional responses in both astrocytes and microglia. Principal component and differential expression analyses demonstrated clear separation between normoxic and hypoxic samples, indicating that hypoxia establishes a dominant transcriptional landscape upon which infection-associated responses occur. These findings are consistent with previous studies demonstrating that chronic hypoxic stress profoundly remodels glial gene expression and may represent a key feature of the ageing and AD brain [78,79].

Despite the dominant effects of hypoxia, *C. pneumoniae* infection induced distinct, lineage-specific transcriptional responses. Under normoxic conditions, astrocytes exhibited relatively modest changes in metabolic gene expression, with no significantly altered genes detected in the metabolic panel. In contrast, several AD-associated genes, including ELOVL1, FKBP4, FN1, TGFBR1, FSTL1 and DLG3, were significantly altered, suggesting that infection preferentially influences pathways involved in extracellular matrix remodeling, cellular stress responses and neuronal support functions. Alterations in FN1 and TGFBR1 may further reflect transcriptional changes associated with tissue remodelling and reactive astrocyte responses, both of which have been implicated in neurodegenerative disease [80,81].

Microglia displayed a different transcriptional profile under normoxic conditions. Similar to astrocytes, no significantly differentially expressed genes were identified in the NanoString Metabolic Panel following *C. pneumoniae* infection. Although several genes exhibited expression changes in the volcano plot using the NanoString nSolver Advanced Analysis threshold, none met the conventional significance threshold (FDR < 0.05). In contrast, significant differential expressions were observed in the NanoString Alzheimer’s Disease Panel, suggesting that under normoxic conditions, *C. pneumoniae* infection preferentially influences Alzheimer’s disease-associated transcriptional pathways rather than core metabolic processes.

Hypoxia markedly amplified transcriptional responses in both glial lineages. In astrocytes, combined hypoxia and *C. pneumoniae* infection induced robust upregulation of genes associated with hypoxia signalling, glucose metabolism and metabolic adaptation, including *HK2*, *PDK1*, *CA9*, *ENO2*, *PFKFB3*, *SLC2A1*, *EPAS1* and *NDRG1*. Collectively, these changes are consistent with a hypoxia-responsive transcriptional program and suggest altered regulation of metabolic pathways in astrocytes under combined stress conditions. In contrast, hypoxic microglia demonstrated induction of hypoxia-responsive genes, including *HIF1A*, *HK2*, *PDK1*, *EPAS1* and *NDRG1*, together with altered expression of lysosomal and metabolic regulators such as *HEXB*, *NEU1*, *RRAGC*, *FNIP1* and *MAP1LC3B*. These findings are consistent with transcriptional alterations in stress-responsive, lysosomal, autophagic and metabolic pathways, suggesting a distinct microglial response to combined hypoxic and infectious stress.

Factorial analysis further demonstrated that the transcriptional response to *C. pneumoniae* infection was dependent on oxygen availability, with significant infection × oxygen interaction effects identified across both NanoString panels and in both glial cell types. Notably, interaction effects were more prevalent within the AD panel than the Metabolic Pathways panel, particularly in astrocytes, indicating that the influence of oxygen availability on the response to infection extended beyond metabolic pathways to a broader range of AD-related genes. Several prominent interaction effects involved genes associated with hypoxia-responsive and metabolic processes, including *HK2*, *CA9*, *SLC2A1*, *PFKFB3*, *NDRG1* and *EPAS1*. Together, these findings provide direct statistical evidence that infection-evoked transcriptional responses differ according to oxygen availability and support the concept that infection and hypoxic stress interact at the transcriptional level in a cell-type-specific manner.

Taken together, these cell-type-specific transcriptional responses highlight the broader molecular consequences of combined infectious and hypoxic stress. Given the central role of glial metabolism in regulating neuroinflammation and amyloid processing, these findings provide further support for the hypothesis that microbial infection and vascular dysfunction may converge to influence disease-relevant pathways in AD.

Proteomic profiling demonstrated that *C. pneumoniae* infection induced substantial remodeling of the glial proteome, although the magnitude and composition of these responses differed markedly between astrocytes and microglia and were modified by oxygen availability. Under normoxic conditions, infection produced extensive alterations in protein abundance in both astrocytes and microglia. In contrast, noticeably fewer proteins were differentially expressed under hypoxic conditions. Factorial analysis provided direct statistical evidence that the infection-associated protein abundance response differed according to oxygen availability for a subset of proteins, with significant oxygen × infection interaction effects identified for 32 of 5338 proteins (0.6%) in astrocytes and 4 of 5062 proteins (0.08%) in microglia. Thus, although oxygen-dependent interaction effects were detectable at the protein level, they were restricted to a relatively small proportion of the quantified proteome.

Although hypoxia markedly amplified transcriptional responses in both astrocytes and microglia, a similarly broad response was not observed at the proteomic level. Instead, infection under hypoxic conditions resulted in no significantly differentially abundant proteins in astrocytes and only a small number in microglia, with neither cell type exhibiting significantly enriched canonical pathways. This distinction was further supported by the factorial analyses. Whereas significant oxygen × infection interactions were widespread across the NanoString datasets, significant proteomic interactions were limited to 32 proteins in astrocytes and 4 proteins in microglia. Thus, oxygen-dependent modulation of the infection response was considerably more extensive at the transcriptional than protein-abundance level at the 72 h time point examined. This divergence between the transcriptomic and proteomic datasets suggests that hypoxia may alter the relationship between transcription and protein synthesis through post-transcriptional regulatory mechanisms. Such transcriptome–proteome discordance has been widely reported during hypoxic stress, during which extensive hypoxia-responsive transcription can occur alongside suppression of global protein synthesis to conserve cellular energy [82]. This translational repression is mediated, in part, through inhibition of mammalian target of rapamycin (mTOR) signalling via the REDD1–TSC1/TSC2 pathway [83] and phosphorylation of the eukaryotic translation initiation factor eIF2α, which selectively limits protein synthesis while permitting translation of stress-responsive transcripts [84]. Consequently, widespread changes in mRNA abundance do not necessarily result in proportional alterations in protein abundance. These established mechanisms provide potential explanations for the discordance observed in the present study; however, HIF-1α protein abundance or activity and translational regulation were not directly assessed. Differences in the temporal dynamics of transcription and protein abundance may also contribute to the observed discordance, as protein-level alterations may emerge over longer periods than the 72 h experimental window examined here.

In astrocytes, *C. pneumoniae* infection altered proteins involved in intracellular signalling, cytoskeletal organisation and immune regulation. Notably, significant reductions in proteins such as A-kinase anchoring protein 9 (AKAP9), cilia- and flagella-associated protein 54 (CFAP54) and complement component C3 suggest disruption of signalling scaffolds, structural homeostasis and innate immune pathways. Conversely, increased abundance of proteins involved in RNA processing and post-transcriptional regulation, including serine/arginine repetitive matrix protein 2 (SRRM2), is consistent with alterations in cellular programmes involved in RNA metabolism and protein homeostasis during intracellular infection. Collectively, these findings indicate extensive astrocytic remodelling in response to *C. pneumoniae* infection, consistent with the phenotypic plasticity exhibited by reactive astrocytes under pathological conditions [85]. Such alterations are also consistent with previous studies demonstrating that *Chlamydia* species extensively exploit host cellular machinery to facilitate intracellular survival, persistence and metabolic adaptation within host cells [86]. 

Microglia exhibited a distinct proteomic signature characterised by alterations in proteins associated with immunometabolic regulation. Increased abundance of ATP citrate lyase (ACLY), a key regulator of lipid metabolism and inflammatory activation, is consistent with alterations in proteins involved in metabolic and immune regulation. In contrast, reduced abundance of metalloproteinase domain-containing protein 19 (ADAM19), matrix metalloproteinase 27 (MMP27) and purinergic receptor P2Y4 (P2RY4) was observed. These proteins have established roles in intercellular communication and immune regulation—features previously associated with dysfunctional microglial states in neurodegenerative disease [87,88,89].

A notable finding was that infection-associated proteomic responses differed according to oxygen availability, although significant factorial interaction effects were restricted to a relatively small subset of proteins. Under hypoxic conditions, relatively few proteins were differentially expressed and no significantly enriched canonical pathways were identified in either glial lineage. These findings indicate that infection-associated proteomic responses differ substantially under hypoxic conditions compared with normoxia. Together with the factorial interaction analysis, these findings suggest that oxygen availability modifies specific components of the infection-associated proteomic response rather than producing the widespread interaction effects observed at the transcriptional level. Altered abundance of proteins involved in lipid metabolism and proteostasis, including very low-density lipoprotein receptor (VLDLR) and proteasome 26S subunit, non-ATPase 1 (PSMD1), further suggests that combined hypoxic and infectious stress may promote cellular dysfunction relevant to Alzheimer’s disease, where dysregulated lipid homeostasis and impaired protein clearance are recognized pathological features [90,91]. Together, these findings indicate that oxygen availability influences glial responses to *C. pneumoniae* infection and may influence disease-relevant pathways associated with neurodegeneration.

Alongside these metabolic and molecular alterations, marked changes in the extracellular amyloid-β peptide profile were observed. Across both astrocytes and microglia, infection was associated with increased Aβ42/Aβ40 ratios, with the greatest ratio observed under combined hypoxia and infection in astrocytes. The Aβ42/Aβ40 ratio is widely recognised as an indicator of relative Aβ peptide composition and has been associated with amyloidogenic potential and aggregation in AD [92,93]. Notably, changes in the Aβ42/Aβ40 ratio were more pronounced than changes in the absolute concentrations of either peptide, indicating that the experimental conditions altered the relative extracellular abundance of Aβ42 and Aβ40.

These findings can be considered in the context of the antimicrobial protection hypothesis of AD, which proposes that Aβ may function as an innate immune effector in response to microbial challenge [94]. The increased Aβ42/Aβ40 ratios observed following *C. pneumoniae* infection are consistent with an association between microbial stress and altered extracellular Aβ peptide balance. Hypoxia further modified this response, particularly in astrocytes, where the highest Aβ42/Aβ40 ratio occurred under combined hypoxia and infection. However, the present experiments did not assess APP cleavage, secretase activity, peptide degradation or clearance and therefore the mechanisms underlying these changes cannot be determined from the current data. Nonetheless, the findings raise the possibility that microbial and hypoxic stress may converge to influence Aβ homeostasis, however direct mechanistic investigation is required.

Together, our findings support a model in which hypoxia establishes a stress-adapted cellular environment that modifies glial responses to microbial infection. Astrocytes exhibited comparatively adaptive metabolic responses, maintaining elevated intracellular ATP despite reduced oxidoreductase activity, whereas microglia displayed greater metabolic vulnerability, characterised by reduced ATP and altered metabolic homeostasis under combined stress. The concurrent alterations in glial metabolism, molecular signalling and extracellular Aβ peptide balance suggest that microbial infection and hypoxic stress may converge on AD-relevant cellular processes. However, the present findings do not establish a causal relationship between metabolic reprogramming and altered Aβ homeostasis or demonstrate a direct contribution to AD pathogenesis.

Several limitations should be acknowledged. The use of immortalized glial cell lines enabled controlled investigation of infection–hypoxia interactions but does not fully recapitulate the complexity of primary human glia or the in vivo brain microenvironment. Accordingly, the observed molecular and functional responses should be interpreted as AD-relevant cellular changes rather than as direct evidence of AD pathogenesis. The present study included three independent biological replicates per experimental condition, which represents a limitation, particularly for high-dimensional transcriptomic and proteomic analyses. The relatively small sample size may reduce statistical power to detect subtle treatment and interaction effects and may increase sensitivity to biological variability.

Furthermore, although the multi-omics approach identified numerous disease-relevant pathways, direct mechanistic relationships between specific molecular alterations and amyloid processing were not experimentally examined. Aβ40 and Aβ42 measurements identified treatment-associated alterations in the extracellular Aβ peptide profile; however, APP cleavage, secretase activity and Aβ degradation or clearance were not assessed, preventing determination of the mechanisms underlying these changes. Similarly, HIF-1α protein abundance or activity was not directly assessed and therefore hypoxia-responsive transcriptional changes cannot be attributed directly to HIF-1α activity. Although 2-NBDG measurements demonstrated alterations in glucose uptake, glycolytic flux was not directly measured and MTT reduction reflects cellular reductive activity rather than providing a direct measure of cell viability or survival.

Infection was confirmed qualitatively by immunofluorescence; however, infection burden, inclusion number and inclusion size were not quantitatively compared between normoxic and hypoxic conditions. Consequently, the possibility that oxygen availability altered *C. pneumoniae* replication or infection dynamics cannot be excluded. Future studies using primary human cells, co-culture systems and in vivo models, together with direct metabolic flux measurements, independent measures of cell viability, quantitative assessment of infection burden and targeted validation of key molecular findings, will be important to determine how chronic infection and hypoxia interact to influence neurodegenerative processes in vivo.

## 4. Materials and Methods

**Figure 14:**
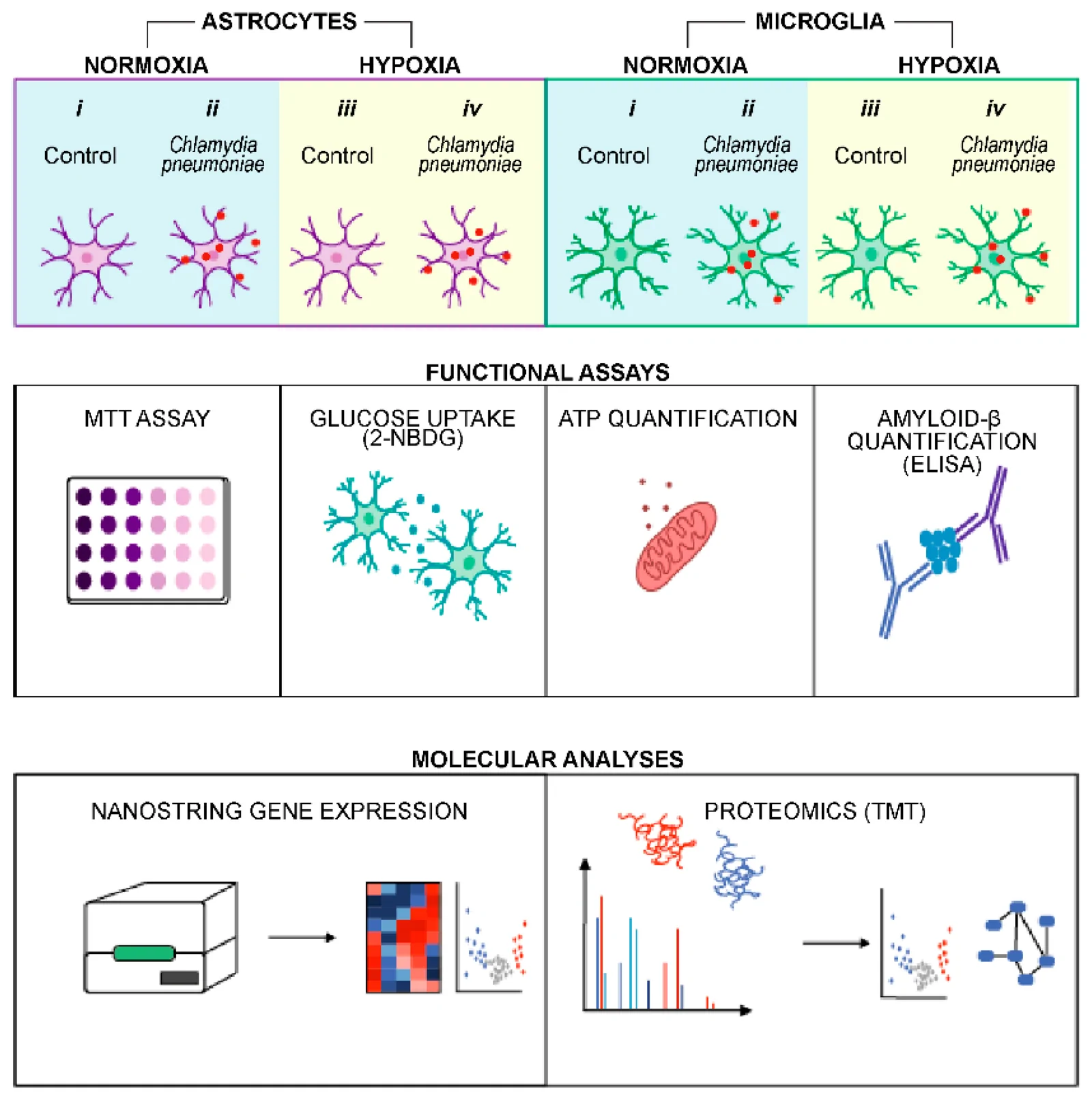
An illustrative overview of the experimental design and analytical workflow. Human astrocytes (SVG p12) and microglia (HMC3) were cultured under four experimental conditions for 72 h: (i) normoxic control (21% O_2_ and 5% CO_2_), (ii) normoxic *C. pneumoniae* infection (MOI = 1), (iii) hypoxic control (3% O_2_, 5% CO_2_ and 92% N_2_) and (iv) combined hypoxia and *C. pneumoniae* infection (MOI = 1, 3% O_2_). Functional assays were performed MTT, glucose uptake (2-NBDG), intracellular ATP levels and amyloid-β secretion (Aβ_40_ and Aβ_42_ ELISA). Molecular profiling included NanoString nCounter gene expression analysis using the Alzheimer’s Disease and Metabolic Pathways panels, together with Tandem Mass Tag (TMT)-based quantitative proteomics. Differential expression and pathway analyses were subsequently performed to characterise the effects of infection, hypoxia and their combined exposure on glial cell metabolism and Alzheimer’s disease-related molecular pathways.

An overview of the experimental workflow is provided in Figure 14 to summarise the study design and the analytical approaches undertaken. Human astrocytes and microglia were cultured under normoxic and hypoxic conditions in the presence or absence of *C. pneumoniae* infection, generating four experimental groups for each cell type. Functional assays were performed to assess MTT reduction, glucose uptake, intracellular ATP levels and amyloid-β secretion, while molecular analyses included NanoString gene expression profiling and Tandem Mass Tag (TMT)-based quantitative proteomics.

### 4.1. Cell Culture

Human astrocytes (SVG p12; ATCC CRL-8621) and human microglia (HMC3; ATCC CRL-3304) were obtained from the American Type Culture Collection (ATCC, Manassas, VA, USA). Cells were maintained in Dulbecco’s Modified Eagle Medium (DMEM; Gibco, Thermo Fisher Scientific, Carlsbad, CA, USA; Cat. No. 11965092) supplemented with 10% fetal bovine serum (FBS; Gibco, Thermo Fisher Scientific, Carlsbad, CA, USA; Cat. No. 10099141) and 1% GlutaMAX (Gibco, Thermo Fisher Scientific, Carlsbad, CA, USA; Cat. No. 35050061). Cultures were maintained at 37 °C in a humidified atmosphere containing 5% CO_2_. Experiments were performed using three independent biological replicates per experimental condition (*n* = 3), with each biological replicate representing an independent experiment conducted on a separate day using cells from a separate passage (<35). Within each oxygen condition, control and infected groups were passage-matched. For functional assays, each biological replicate was assessed in technical triplicate, with technical replicates averaged within each biological replicate prior to statistical analysis. NanoString and TMT-based proteomic analyses were performed using three independent biological replicates per experimental condition. The use of three biological replicates per condition was selected to balance biological replication with the scale and resource requirements of the multi-assay and multi-omics experimental design.

Biological replicates were generated independently for the different experimental assays; therefore, the same biological samples were not necessarily used across functional, NanoString and proteomic analyses. Astrocytic and microglial phenotypes were confirmed by immunofluorescence staining for GFAP and Iba1, respectively. Cultures were also visually assessed using Hoechst staining for extranuclear punctate DNA staining suggestive of mycoplasma contamination, with no characteristic staining observed. Formal genetic cell-line authentication and molecular mycoplasma testing were not performed.

### 4.2. Hypoxia and Combined Infection-Hypoxia Conditions

Experimental conditions comprised four treatment groups: (i) normoxic control, (ii) *C. pneumoniae* infection under normoxia, (iii) hypoxia control and (iv) combined hypoxia and *C. pneumoniae* infection. Hypoxic cultures were maintained in a humidified tri-gas incubator at 3% O_2_, 5% CO_2_ and 92% N_2_ at 37 °C for 72 h, whereas normoxic controls were maintained at 21% O_2_ and 5% CO_2_ under identical temperature and humidity conditions. The 3% O_2_ condition was selected to model physiologically relevant hypoxia based on reported reductions in brain tissue oxygen levels below approximately 4% O_2_ during hypoxic conditions and previous in vitro studies demonstrating hypoxia-associated cellular responses in glial cells at oxygen concentrations of 1–5% [95]. The 72 h exposure period corresponded to the duration of *C. pneumoniae* infection, enabling the effects of infection and hypoxia to be assessed over an equivalent experimental period. For hypoxia-only experiments, uninfected cells were transferred to hypoxic conditions immediately following cell seeding. For combined hypoxia and infection experiments, cells were infected with *C. pneumoniae* at a multiplicity of infection (MOI) of 1 before immediate transfer to the hypoxic incubator, where they remained for the duration of the experiment.

### 4.3. Chlamydia pneumoniae Infection Model

Astrocytes and microglia were cultured to approximately 70–80% confluence before infection. Cells were infected with *Chlamydia pneumoniae* strain AR-39 (ATCC 53592), originally isolated from a human throat and propagated in HEp-2 cells (ATCC CCL-23) before expansion in BEAS-2B cells. The infectious titre of the expanded stock had previously been determined by serial dilution and quantification of inclusion-forming units (IFU), and this established titre was used to standardise infections at an MOI = 1. Following infection, cultures were maintained under their respective oxygen conditions for 72 h before downstream analyses.

### 4.4. Immunofluorescence Microscopy

For immunofluorescence analyses, astrocytes and microglia were cultured in black-walled, clear-bottom 96-well plates and fixed with 4% (*w*/*v*) paraformaldehyde for 15 min at room temperature. Cells were permeabilized with 0.3% Triton™ X-100 and blocked with 1% (*w*/*v*) bovine serum albumin (BSA) prior to incubation with primary antibodies against GFAP for astrocytes, Iba1 for microglia and *Chlamydia trachomatis* lipopolysaccharide (LPS), which is reported by the manufacturer to cross-react with *C. pneumoniae*. Species-appropriate Alexa Fluor™-conjugated secondary antibodies was subsequently applied and nuclei were counterstained with Hoechst 33342. Details of primary and secondary antibodies, including suppliers, catalogue numbers and working dilutions, are provided in Table A1. Fluorescence images were acquired using an IXplore SpinSR spinning-disk confocal microscope (Evident, Tokyo, Japan) under identical acquisition settings within each experiment and processed using cellSens software v4.3 (Evident).

Imaging was performed using a predefined region of interest positioned consistently within each well. At 30× magnification, images were acquired as a 5 × 5 tiled field comprising 25 contiguous frames, while 100× imaging was performed as a 7 × 7 tiled field comprising 49 contiguous frames. Individual frames were stitched to generate larger composite images. Microscopy was used for qualitative confirmation of cellular phenotype and intracellular *C. pneumoniae* localisation rather than quantitative image analysis or scoring; therefore, blinded image analysis was not performed.

### 4.5. MTT Reduction Assay

Cellular reductive activity was assessed using the MTT Cell Proliferation Assay 3-(4,5-dimethylthiazol-2-yl)-2,5-diphenyltetrazolium bromide; Invitrogen, Thermo Fisher Scientific, Eugene, OR, USA; Cat. No. V13154). Astrocytes and microglia were seeded into 96-well plates at a density of 4.0 × 10^3^ cells per well and cultured overnight before treatment. Following exposure to the experimental conditions described above, 10 µL of PBS (PBS; Gibco, Thermo Fisher Scientific, Carlsbad, CA, USA; Cat. No. 10010023) was added to each well to achieve a final concentration of 0.5 mg/mL. Cells were incubated for 3 h at 37 °C, after which the medium was removed, and the resulting formazan crystals were dissolved in 100 µL dimethyl sulfoxide (DMSO). Plates were shaken at 400 rpm for 10 min before absorbance was measured at 570 nm with a reference wavelength of 630–690 nm using a POLARstar Omega microplate reader (BMG LABTECH, Ortenberg, Germany). Blank wells containing culture medium without cells were included for background correction.

### 4.6. Glucose Uptake Assay

Glucose uptake was quantified using the 2-NBDG Glucose Uptake Assay Kit [2-(N-(7-nitrobenz-2-oxa-1,3-diazol-4-yl)amino)-2-deoxyglucose] (Invitrogen, Thermo Fisher Scientific, Eugene, OR, USA; Cat. No. N13195) according to the manufacturer’s instructions. Following exposure to the experimental conditions described above, cells were washed twice with DPBS and incubated with 100 µM 2-NBDG in glucose-free medium for 30 min at 37 °C. Excess dye was removed by washing with DPBS and fluorescence was measured using a POLARstar Omega microplate reader (BMG LABTECH, Ortenberg, Germany) at excitation and emission wavelengths of 485 and 535 nm, respectively. Fluorescence values were normalised to cell number determined from parallel wells to account for differences in cell density. Data are presented as the mean ± SEM from three independent biological replicates.

### 4.7. ATP Quantification

Intracellular ATP levels were quantified using the Luminescent ATP Detection Assay Kit (Abcam, Cambridge, UK; Cat. No. ab113849) according to the manufacturer’s instructions. Following exposure to the experimental conditions described above, culture medium was removed and cells were washed once with DPBS before addition of the ATP reaction mixture. Following a 5 min incubation at room temperature in the dark, luminescence was measured using a POLARstar Omega microplate reader (BMG LABTECH, Ortenberg, Germany). ATP concentrations were calculated from a standard curve (0–10 µM ATP) and normalised to cell number determined from parallel wells by immunofluorescence microscopy. Data are presented as the mean ± SEM from three independent biological replicates.

### 4.8. Amyloid-β Quantification

Amyloid-β peptide concentrations in cell culture supernatants were quantified using the Human Amyloid Beta (Aβ) 1–40 ELISA Kit (Abcam, Cambridge, UK; Cat. No. ab193692) and the Human Amyloid Beta (Aβ) 1–42 Ultrasensitive ELISA Kit (Thermo Fisher Scientific, Waltham, MA, USA; Cat. No. KHB3544), according to the manufacturers’ instructions. Standards and samples were analysed in duplicate. Absorbance was measured at 450 nm using a POLARstar Omega microplate reader (BMG LABTECH, Ortenberg, Germany). Aβ_40_ and Aβ_42_ concentrations were calculated from standard curves and normalised to cell number determined from parallel wells, with values expressed as pg/1000 cells. The Aβ_42_/Aβ_40_ ratio was calculated from the corresponding measured peptide concentrations.

### 4.9. NanoString Gene Expression Profiling

Total RNA was extracted from astrocyte and microglia cultures exposed to four experimental conditions (control, *C. pneumoniae* infection, hypoxia and combined hypoxia with *C. pneumoniae* infection; *n* = 3 biological replicates per condition) using the RNeasy Mini Kit (QIAGEN catalogue number: 74104, Hilden, Germany) according to the manufacturer’s instructions. RNA was eluted in 40 µL of nuclease-free water and RNA concentration and purity were determined using a NanoDrop ND-1000 spectrophotometer (Thermo Fisher Scientific, Wilmington, DE, USA). RNA integrity was confirmed prior to downstream analyses.

Gene expression profiling was performed using the NanoString nCounter Analysis System (NanoString Technologies, Seattle, WA, USA) with the nCounter Alzheimer’s Disease Panel (Cat. No. XT-CSO-MAD1-12) and the nCounter Metabolic Pathways Panel (Cat. No. XT-CSO-HSMP1-12). The Alzheimer’s Disease Panel quantifies 770 genes representing 23 biological processes associated with Alzheimer’s disease together with 10 internal housekeeping genes, whereas the Metabolic Pathways Panel quantifies 771 genes involved in central metabolic processes, including glycolysis, oxidative phosphorylation, lipid metabolism and mitochondrial function.

Hybridisation reactions were prepared according to the manufacturer’s protocol using 125 ng of total RNA per sample and incubated at 65 °C for 20 h. Hybridised samples were processed using the nCounter^®^ Prep Station (NanoString Technologies, Inc. Seattle, WA, USA) before digital barcode quantification on the nCounter Digital Analyzer (NanoString Technologies, Inc. Seattle, WA, USA) using the high-sensitivity setting (555 fields of view).

### 4.10. NanoString Data Analysis

Raw NanoString nCounter gene expression data were analysed using nSolver Analysis Software (NSolver 4.0 (noting R version R 3.3.2 needed)) with the Advanced Analysis Module (NanoString Technologies, Seattle, WA, USA). Quality control, normalisation and differential expression analyses were performed using the default Advanced Analysis workflow, including automatic filtering of low-count genes according to the manufacturer’s recommended thresholds. Differential expression analyses were conducted separately for each experimental comparison by specifying the relevant experimental condition as the predictor variable, including infected versus control under normoxia, infected versus control under hypoxia, hypoxia versus normoxia in control cells and hypoxia versus normoxia in infected cells. Statistical significance was assessed using the fast approximation of the Benjamini–Yekutieli FDR correction implemented within the Advanced Analysis Module.

Volcano plots and differential expression statistics were generated for both the Alzheimer’s Disease and Metabolic Pathways panels using nSolver Advanced Analysis. Pathway score analyses and pathway heatmaps were generated for the Metabolic Pathways Panel using the integrated pathway scoring workflow. Full differential gene datasets for each experimental contrast are provided in Appendix A for the metabolic panel and Appendix B for the Alzheimer’s disease panel.

To directly assess infection × oxygen interaction effects, an additional factorial analysis was performed separately for astrocytes and microglia for each NanoString panel using the limma package in R (version 4.5.0; R Foundation for Statistical Computing, Vienna, Austria). Log_2_-transformed normalised expression values were modelled as a function of oxygen condition, infection status and their interaction (Expression ~ Oxygen × Infection). The interaction coefficient represented the difference in the effect of infection between hypoxic and normoxic conditions. Multiple testing was controlled using the Benjamini–Hochberg procedure, with FDR < 0.05 considered statistically significant. Full interaction statistics are provided in Supplementary Tables S6–S9.

PCA was performed separately for the Alzheimer’s Disease and Metabolic Pathways panels in R (version 4.5.0; R Foundation for Statistical Computing, Vienna, Austria) using log_2_-transformed normalised expression values exported from nSolver. PCA was used to evaluate global transcriptional variation and sample clustering across experimental groups.

### 4.11. Tandem Mass Tag (TMT) Quantitative Proteomic Profiling

To complement the transcriptomic analyses, quantitative Tandem Mass Tag (TMT)-based proteomic profiling was performed to characterise infection-associated changes in protein abundance in astrocytes and microglia cultured under normoxic and hypoxic conditions. Protein extracts were prepared from all experimental groups (*n* = 3 biological replicates per condition).

Cells were lysed in radioimmunoprecipitation assay (RIPA) buffer supplemented with protease and phosphatase inhibitor cocktails. Lysates were sonicated on ice and centrifuged at 14,000× *g* for 15 min at 4 °C to remove cellular debris. Supernatants were collected and total protein concentrations were determined using the Pierce™ Bicinchoninic Acid (BCA) Protein Assay Kit (Thermo Fisher Scientific, Rockford, IL, USA) according to the manufacturer’s instructions.

Proteomic analyses were performed by Proteomics International (Perth, WA, Australia). Briefly, 100 µg of total protein from each sample was reduced, alkylated and digested overnight with sequencing-grade modified trypsin (Promega, Madison, WI, USA). Peptides were labelled using TMT 10-plex reagents (Thermo Fisher Scientific, Rockford, IL, USA) with each biological replicate individually labelled in a separate TMT channel before the labelled samples were pooled into multiplexed samples, thereby retaining individual biological replicate information. Peptide mixtures were fractionated by high-pH reversed-phase chromatography and analysed by nano-liquid chromatography tandem mass spectrometry (nanoLC-MS/MS) using a Q Exactive™ HF Hybrid Quadrupole-Orbitrap™ Mass Spectrometer (Thermo Fisher Scientific, Bremen, Germany).

### 4.12. Proteomic Data Analysis

Raw mass spectrometry data were processed using Proteome Discoverer™ (version 2.4; Thermo Fisher Scientific, Bremen, Germany) with the Sequest HT search engine against the SwissProt Homo sapiens protein database (August 2024; 20,582 sequences). Database searches were performed using trypsin specificity with a maximum of two missed cleavages, carbamidomethylation of cysteine as a fixed modification and oxidation of methionine as a variable modification. Peptide and protein identifications were filtered using a target-decoy strategy with a FDR of <1% at both the peptide and protein levels.

Protein-level quantification was performed using the proteinSummarization function of the MSstatsTMT package (MsstatsTMT_2.14.2) in R. Peptide-level intensities generated by Proteome Discoverer were summarised to protein-level abundances using the MSstats model-based approach. Missing values were imputed using the default MSstats imputation method and global normalisation was applied to account for overall intensity differences between TMT channels. Reference-channel normalisation was not applied and channels designated as empty or for normalisation were excluded prior to protein summarisation. No additional batch correction was performed.

Differential protein abundance analyses were performed for each experimental comparison using the limma package in R. A linear model was fitted to each protein using lmFit, specified pairwise contrasts were applied using contrasts.fit and empirical Bayes moderation was performed using eBayes to generate moderated t-statistics, log_2_ fold changes, raw *p*-values and adjusted *p*-values. Multiple testing was controlled using the Benjamini–Hochberg procedure, with an FDR < 0.05 considered statistically significant. Volcano plots display log_2_ fold change against −log_10_(*p*-value), with proteins meeting both FDR < 0.05 and |log_2_ fold change| > 0.58 highlighted as significantly altered.

Functional interpretation of significantly differentially abundant proteins was undertaken using Ingenuity Pathway Analysis (IPA; QIAGEN, Hilden, Germany). Proteins meeting an FDR < 0.05 were included in IPA Core Analysis together with their log_2_ fold change and associated statistical values. Core analyses were performed using log_2_ fold change values and associated statistical values to identify enriched canonical pathways, upstream regulators and predicted activation or inhibition states. Canonical pathway enrichment was assessed using right-tailed Fisher’s exact tests, with *p* < 0.05 considered significant. IPA activation z-scores ≥ 2 or ≤−2 were interpreted as predicted activation or inhibition, respectively. A minimum of five proteins per pathway was required for pathway enrichment to be reported. Differential protein datasets for all experimental comparisons are provided in Supplementary Material.

To determine whether infection-associated changes in protein abundance differed according to oxygen availability, an additional 2 × 2 factorial analysis was performed using the normalised protein-level abundances generated by the MSstatsTMT pipeline. Linear models incorporating oxygen condition, infection status, and the oxygen × infection interaction were fitted separately for astrocytes and microglia using limma. The interaction term represented the difference in the infection-associated protein abundance response between hypoxic and normoxic conditions, calculated as (hypoxic infected − hypoxic control) − (normoxic infected − normoxic control). Multiple testing was controlled using the Benjamini–Hochberg procedure, with FDR < 0.05 considered statistically significant.

## 5. Conclusions

This study demonstrates that *C. pneumoniae* infection and hypoxia interact to alter glial metabolic and molecular responses in human astrocyte and microglial cell models. Combined exposure was associated with cell-type-specific changes in bioenergetics, transcriptional remodeling of pathways related to mitochondrial function, lipid metabolism and neurodegeneration and alterations in the extracellular amyloid-β peptide profile, including an increased Aβ42/40 ratio. These findings support the concept that microbial infection and vascular stressors may converge to influence glial function and pathways relevant to AD. While the use of immortalized cell lines limits direct translation to human disease, the results provide insight into how infection and hypoxia may contribute to neurodegeneration-associated cellular dysfunction and identify potential targets for future investigation in more physiologically relevant models.

## Data Availability

The raw data supporting the conclusions of this article will be made available by the authors on request.

## Supplementary Materials

The following supporting information can be downloaded at: https://www.mdpi.com/article/10.3390/ijms27198820/s1.

## Abbreviations

The following abbreviations are used in this manuscript:

Aβ	Amyloid-β
AD	Alzheimer’s Disease
APOE	Apolipoprotein E
ATP	Adenosine triphosphate
CNS	Central nervous system
CPAF	*Chlamydial* Protease-Like Activity Factor
*C. pneumoniae*	*Chlamydia pneumoniae*
HIF	Hypoxia-inducible factor
MOI	Multiplicity of infection
mTOR	Mammalian target of rapamycin
OXPHOS	Oxidative phosphorylation
PCA	Principal component analysis
ROS	Reactive oxygen species
TMT	Tandem mass tag

## Appendix A. Immunofluorescence Specifics

### Primary and Secondary Antibodies

**Table A1:** Primary and Secondary Antibodies.

Target	Supplier/Catalogue No.	Dilution
*Chlamydia* LPS ^1^	Abcam/ab106853	1:500
Anti-GFAP	Abcam/ab4674	1:600
Anti-Iba1	Abcam/ab318302	1:200
Donkey anti-Goat Alexa Fluor™ 647	Thermo Fisher Scientific/A-21447	1:600
Donkey anti-Chicken Alexa Fluor™ 488	Thermo Fisher Scientific/A748948	1:600
Hoechst	-	1:2000

^1^ Although raised against *Chlamydia trachomatis*, this antibody cross-reacts with *Chlamydia pneumoniae* owing to the conserved lipopolysaccharide (LPS) epitope shared between *Chlamydia* species.

## Appendix B. NanoString Differentially Expressed Genes

### Appendix B.1. Differentially Expressed Genes Derived from the NanoString Metabolic Panel

**Table A2:** Top 10 significantly differentially expressed genes from the NanoString Metabolic Panel for the comparison of *C. pneumoniae*-infected and control astrocytes under hypoxic conditions.

Gene	Log_2_ Fold Change	*p*-Value	FDR
*DTL*	1.02	5.89 × 10^−7^	0.00218
*SERINC1*	0.79	1.39 × 10^−6^	0.00257
*SLC2A1*	2.81	3.38 × 10^−6^	0.0026
*SLC2A14*	3.15	3.40 × 10^−6^	0.0026
*FDXR*	3.45	4.08 × 10^−6^	0.0026
*GAPDH*	1.33	4.81 × 10^−6^	0.0026
*MYB*	1.45	4.93 × 10^−6^	0.0026
*HK2*	3.85	6.64 × 10^−6^	0.00307
*ALDOA*	1.84	1.01 × 10^−5^	0.00373
*NFKB1*	1.26	1.29 × 10^−5^	0.00392

**Table A3:** Top 10 significantly differentially expressed genes from the NanoString Metabolic Panel for the comparison of *C. pneumoniae*-infected and control microglia under hypoxic conditions.

Gene	Log_2_ Fold Change	*p*-Value	FDR
*MAP1LC3B*	1.52	3.39 × 10^−5^	0.0405
*SLC2A14*	2.01	3.45 × 10^−5^	0.0405
*RB1CC1*	0.713	3.69 × 10^−5^	0.0405
*NEU1*	1.36	5.05 × 10^−5^	0.0405
*ACAP2*	0.285	7.80 × 10^−5^	0.0405
*HK2*	2.15	8.71 × 10^−5^	0.0405
*RRAGC*	1.56	9.82 × 10^−5^	0.0405
*UPP1*	2.51	1.00 × 10^−4^	0.0405
*SLC7A5*	1.99	1.22 × 10^−4^	0.0405
*GOT2*	−0.47	1.29 × 10^−4^	0.0405

**Table A4:** Top 10 differentially expressed genes from the NanoString Metabolic Panel for the comparison of hypoxia and normoxia in control astrocytes.

Gene	Log_2_ Fold Change	*p*-Value	FDR
NDUFA1	−0.952	6.82 × 10^−6^	0.013
*BHMT2*	−4.34	8.12 × 10^−6^	0.013
*LDHC*	−3.29	1.20 × 10^−5^	0.013
*LDHB*	−0.859	2.00 × 10^−5^	0.013
*HK1*	1.24	2.70 × 10^−5^	0.013
*IDH1*	−0.507	2.75 × 10^−5^	0.013
*MLYCD*	0.943	3.04 × 10^−5^	0.013
*HK2*	0.838	4.03 × 10^−5^	0.013
*JAK2*	−1.47	4.39 × 10^−5^	0.013
*RRAGC*	1.5	4.57 × 10^−5^	0.013

**Table A5:** Top 10 differentially expressed genes from the NanoString Metabolic Panel for the comparison of hypoxia and normoxia in *C. pneumoniae*-infected astrocytes.

Gene	Log_2_ Fold Change	*p*-Value	FDR
*CD63*	1.6	7.04 × 10^−8^	0.000202
*SERINC3*	1.19	1.07 × 10^−7^	0.000202
*PDK3*	3.21	2.27 × 10^−7^	0.000286
*SLC3A1*	−3.09	3.57 × 10^−7^	0.000336
*FNIP1*	2.57	7.14 × 10^−7^	0.000458
*PDK1*	5.05	7.75 × 10^−7^	0.000458
*CTSL*	1.98	9.41 × 10^−7^	0.000458
*ALOX5*	3.29	9.70 × 10^−7^	0.000458
*PLK1*	−3.5	1.47 × 10^−6^	0.000552
*PFKM*	−1.22	1.50 × 10^−6^	0.000552

**Table A6:** Top 10 differentially expressed genes from the NanoString Metabolic Panel for the comparison of hypoxia and normoxia in control microglia.

Gene	Log_2_ Fold Change	*p*-Value	FDR
*PDGFRB*	−1.21	5.40 × 10^−6^	0.0122
*BIRC3*	4.4	8.79 × 10^−6^	0.0122
*PNP*	1.03	1.97 × 10^−5^	0.0122
*PGM2*	1.01	2.00 × 10^−5^	0.0122
*ACACA*	−1.17	2.00 × 10^−5^	0.0122
*PFKM*	−0.633	2.06 × 10^−5^	0.0122
*MYBL1*	0.585	3.83 × 10^−5^	0.0194
*RB1CC1*	−0.889	5.54 × 10^−5^	0.0246
*ERN1*	0.92	8.14 × 10^−5^	0.0321
*HEXA*	0.806	9.22 × 10^−5^	0.0322

**Table A7:** Top 10 differentially expressed genes from the NanoString Metabolic Panel for the comparison of hypoxia and normoxia in *C. pneumoniae*-infected microglia.

Gene	Log_2_ Fold Change	*p*-Value	FDR
*FNIP1*	2.03	3.38 × 10^−6^	0.00464
*HK2*	2.99	3.44 × 10^−6^	0.00464
*NEU1*	1.99	3.75 × 10^−6^	0.00464
*HEXB*	1.94	6.18 × 10^−6^	0.00573
*PNP*	2.25	1.26 × 10^−5^	0.00669
*HIF1A*	−1.71	1.28 × 10^−5^	0.00669
*GLRX*	2.05	1.28 × 10^−5^	0.00669
*MAP1LC3B*	2.41	1.44 × 10^−5^	0.00669
*RRAGC*	2.28	1.98 × 10^−5^	0.00815
*PDPK1*	0.986	2.50 × 10^−5^	0.00862

### Appendix B.2. Differentially Expressed Genes Derived from the NanoString Alzheimer’s Disease Panel

**Table A8:** Top 10 significantly differentially expressed genes from the NanoString Alzheimer’s Disease Panel for the comparison of *C. pneumoniae*-infected and control astrocytes under normoxic conditions.

Gene	Log_2_ Fold Change	*p*-Value	FDR
*ELOVL1*	−0.491	1.42 × 10^−6^	0.00341
*DLG3*	0.296	1.71 × 10^−6^	0.00341
*FN1*	−0.969	3.65 × 10^−6^	0.00484
*FKBP4*	−0.581	5.46 × 10^−6^	0.00543
*HLTF*	0.32	1.37 × 10^−5^	0.0109
*FSTL1*	−0.407	1.92 × 10^−5^	0.0127
*SOX9*	−0.873	3.96 × 10^−5^	0.0225
*TRPC1*	0.301	6.15 × 10^−5^	0.0231
*TGFBR1*	−0.799	6.37 × 10^−5^	0.0231
*NPC1*	−0.32	6.37 × 10^−5^	0.0231

**Table A9:** Top 10 significantly differentially expressed genes from the NanoString Alzheimer’s Disease Panel for the comparison of *C. pneumoniae*-infected and control astrocytes under hypoxic conditions.

Gene	Log_2_ Fold Change	*p*-Value	FDR
*ENO2*	3.03	2.45 × 10^−6^	0.00565
*NDRG1*	5.25	2.87 × 10^−6^	0.00565
*JMJD6*	1.46	3.80 × 10^−6^	0.00565
*DECR1*	−1.47	6.92 × 10^−6^	0.00771
*YWHAG*	1	1.88 × 10^−5^	0.0168
*TMEM144*	1.72	2.30 × 10^−5^	0.0171
*PFKFB3*	3.44	2.68 × 10^−5^	0.0171
*SLC2A1*	2.52	3.28 × 10^−5^	0.0172
*EPAS1*	2.27	3.47 × 10^−5^	0.0172
*TP53BP2*	1.48	4.09 × 10^−5^	0.0182

**Table A10:** Top 10 significantly differentially expressed genes from the NanoString Alzheimer’s Disease Panel for the comparison of *C. pneumoniae*-infected and control microglia under normoxic conditions.

Gene	Log_2_ Fold Change	*p*-Value	FDR
*HSPH1*	0.647	8.28 × 10^−7^	0.00222
*MAPK6*	1.05	1.10 × 10^−6^	0.00222
*FAM49A*	0.738	2.47 × 10^−6^	0.00334
*RAB3A*	1.27	4.86 × 10^−6^	0.00493
*MGST3*	0.274	6.68 × 10^−5^	0.00542
*JMJD6*	0.986	8.19 × 10^−5^	0.00554
*ZFP36*	1.01	1.36 × 10^−5^	0.00712
*COX7A2L*	0.54	1.53 × 10^−5^	0.00712
*PDE1C*	−0.379	1.58 × 10^−5^	0.00712
*ZBTB33*	0.687	2.21 × 10^−5^	0.0084

**Table A11:** Top 10 significantly differentially expressed genes from the NanoString Alzheimer’s Disease Panel for the comparison of *C. pneumoniae*-infected and control microglia under hypoxic conditions.

Gene	Log_2_ Fold Change	*p*-Value	FDR
*PDE1C*	−2.26	7.08 × 10^−6^	0.0179
*ACP2*	−0.974	1.19 × 10^−5^	0.0179
*FAM107B*	2.24	1.66 × 10^−5^	0.0179
*NDRG1*	4.67	1.67 × 10^−5^	0.0179
*WAC*	−0.49	4.80 × 10^−5^	0.0412
*ATG4C*	−1.11	6.72 × 10^−5^	0.0475
*EPAS1*	2.08	8.74 × 10^−5^	0.0475
*LHFPL2*	0.599	9.15 × 10^−5^	0.0475
*ABCA8*	−1.49	1.02 × 10^−4^	0.0475
*MAPK6*	0.794	1.11 × 10^−4^	0.0475
