# Supplementary material for: Combined Effects of Chlamydia pneumoniae Infection and Hypoxia on Glial Metabolism and Alzheimer’s Disease-Related Pathways
Source: Int J Mol Sci. 2026 Oct 2;27(19):8820. doi: 10.3390/ijms27198820 (PMC13665377; doi:10.3390/ijms27198820)
Supplement: Supplementary file 1 [file ijms-27-08820-s001.zip › ijms-4509408-supplementary.pdf]

Table S1. Top 10 significantly differentially abundant proteins identified in astrocytes following *C. pneumoniae* infection under normoxic conditions.

Protein	Log_2 Fold Change	P-Value	FDR
AFP	-3.49	1.4E-11	0.00000021
AKAP9	-4.65	4.8E-10	0.0000024
PPARD	-2.54	4.1E-10	0.0000024
PTPRH	-3.63	3.8E-09	0.000014
MAGEE1	-2.74	6.4E-09	0.000019
MRPS31	-2.56	8.4E-09	0.000021
C3	-3.23	0.000000021	0.000044
CFAP54	-3.28	0.000000023	0.000044
ALB	-3.95	0.000000031	0.000053
UAP1	-1.7	0.000000056	0.000077

Table S2. Proteins contributing to significantly enriched canonical pathways in astrocytes following *C. pneumoniae* infection under normoxic conditions.

Canonical Pathways	Proteins Involved (≥ 5 proteins per pathway)
Acute Phase Response Signaling	ALB, C3, C9, F2, MAPK8, FKB2, SERPIND1, SERPINF1, TF
Hematoma Resolution Signaling Pathway	ALB, APOC2, APOM, C3, C9, GC, NFKB2, SERPINF1, TF
DHCR24 Signaling pathway	ALB, APOC2, APOM, C3, C9, GC, NFKB2, SERPINF1, TF
LXR/RXR Activation	ALB, APOC2, APOM, C3, C9, GC, NFKB2, SERPINF1, TF
IL-12 Signaling and Production in Macrophages	ALB, APOC2, APOM, C3, MAPK8, NFKB2, THBS1
Regulation of Insulin-like Growth Factor (IGF) Transport and Uptake by IGFBPS	ALB, AFP, C3, F2, LAMB1, SERPIND1, TF
Post-Translational Protein Phosphorylation	ALB, AFP, C3, LAMB1, SERPIND1, TF
Germ Cell-Sertoli Cell Junction Signaling	ACTN1, CTNNA1, IQGAP1, JUP, MAPK8, MYO7A
Sertoli Cell-Sertoli Cell Junction Signaling	ACTN1, ARPC5L, CTNNA1, JUP, LAMB1, MAPK8, NFKB2
Sertoli Cell-Germ Cell Junction Signaling Pathway (enhanced)	ARPC5L, CTNNA1, IQGAP1, JUP, LAMB1, MAPK8, NFKB2

Table S3. Top 10 significantly differentially abundant proteins identified in microglia following *C. pneumoniae* infection under normoxic conditions.

Protein	Log_2 Fold Change	P-Value	FDR
ADAM19	-3.31	2.2E-09	0.000028
NXPE2	-4.19	6.9E-09	0.000028
TMOD2	-2.17	5.5E-09	0.000028
ZNF518B	-2.5	8.1E-09	0.000028
TNFAIP8L2	-2.4	0.000000025	0.000068
NELFE	-3.25	0.000000031	0.000071
DDX50	-1.91	0.000000037	0.000073
CRYZ	-1.77	0.000000055	0.000093
SERPINF1	-2.27	0.000000073	0.00011
C1orf115	-1.75	0.000000095	0.00013

Table S4. Proteins contributing to significantly enriched canonical pathways in microglia following *C. pneumoniae* infection under normoxic conditions.

Canonical Pathways	Proteins Involved (≥ 5 proteins per pathway)
Post-Translational Protein Phosphorylation	AFP, ALB, APOE, C3, FGA, MELTF, SERPINA1
Regulation of Insulin-like Growth Factor (IGF) Transport and Uptake by IGFBPS	AFP, ALB, APOE, C3, FGA, MELTF, SERPINA1
DHCR24 Signaling pathway	ALB, APOE, APOH, APOM, C3, FGA, SERPINA1, SERPINF
Acute Phase Response Signaling	ALB, APOH, C3, FGA, SERPIN1A, SERPINF1
IL-12 Signaling and Production in Macrophages	ALB, APOE, APOM, C3, SERPIN1A
LXR/RXR Activation	ALB, APOE, APOH, APOM, C3, FGA, SERPINA1, SERPINF1

Table S5. All significantly differentially abundant proteins identified in microglia following *C. pneumoniae* infection under hypoxic conditions.

Protein	Log_2 Fold Change	P-Value	FDR
FBL	1.18	0.0000058	0.033
PSMD1	1.32	0.0000075	0.033
VLDLR	-1.11	0.000006	0.033
FAHD2A	1.03	0.000015	0.036

UBXN1	-0.93	0.000016	0.036
ZC3H12B	-1.04	0.000014	0.036
SPTAN1	0.97	0.000026	0.049

Table S6. Top 15 significant oxygen × infection interactions in astrocytes identified in the metabolic NanoString panel

Gene	Log_2 Fold Change	P-Value	FDR
HK2	3.81	2.50E-09	1.87E-06
PLK1	-2.72	5.71E-09	2.14E-06
SLC2A14	3.17	1.60E-08	3.99E-06
TFRC	2.95	4.53E-08	5.99E-06
CA9	5.56	4.69E-08	5.99E-06
FNIP2	2.09	5.40E-08	5.99E-06
TRAF1	3.26	5.60E-08	5.99E-06
ERN1	2.73	7.03E-08	6.57E-06
FNIP1	2.29	1.06E-07	8.35E-06
PDK1	3.06	1.12E-07	8.35E-06
AMPD3	2.91	1.91E-07	1.30E-05
RRAGC	1.67	2.52E-07	1.57E-05
PGK1	2.68	2.81E-07	1.57E-05
SLC2A1	2.58	3.08E-07	1.57E-05
FDXR	2.75	3.14E-07	1.57E-05

Table S7. Top 15 significant oxygen × infection interactions in microglia identified in the metabolic NanoString panel

Gene	Log_2 Fold Change	P-Value	FDR
SLC2A14	2.00	2.06E-07	8.63E-05
HK2	2.15	2.36E-07	8.63E-05
FNIP1	1.66	3.46E-07	8.63E-05
SERINC5	2.58	4.67E-07	8.73E-05
UPP1	2.40	6.19E-07	9.27E-05
ERN1	2.84	1.60E-06	1.99E-04
RB1CC1	1.18	3.54E-06	3.79E-04
SLC7A5	1.80	4.10E-06	3.83E-04

RRAGC	1.39	5.03E-06	4.14E-04
NEU1	1.35	5.53E-06	4.14E-04
XDH	3.11	6.93E-06	4.71E-04
VEGFA	2.58	9.73E-06	6.06E-04
AMPD3	2.67	1.10E-05	6.19E-04
PNP	1.13	1.16E-05	6.19E-04
MAP1LC3B	1.55	1.37E-05	6.83E-04

Table S8. Top 15 significant oxygen × infection interactions in astrocytes identified in the Alzheimer's disease NanoString panel

Gene	Log_2 Fold Change	P-Value	FDR
NDRG1	5.53	3.65E-11	2.78E-08
ENO2	3.07	4.98E-10	1.90E-07
SLC2A1	3.04	2.47E-09	6.26E-07
PFKFB3	3.80	4.59E-09	8.74E-07
NPC1	1.70	2.34E-08	2.84E-06
YBX3	2.15	2.34E-08	2.84E-06
EPAS1	2.34	2.61E-08	2.84E-06
DECR1	-1.53	3.82E-08	3.40E-06
IGF1R	1.81	4.01E-08	3.40E-06
YWHAG	1.31	5.79E-08	4.33E-06
FAM107B	2.06	6.28E-08	4.33E-06
JMJD6	1.55	6.82E-08	4.33E-06
MYO1E	2.07	1.86E-07	1.09E-05
TP53BP2	1.49	2.46E-07	1.34E-05
RELL1	1.38	3.38E-07	1.72E-05

Table S9. Top 15 significant oxygen × infection interactions in microglia identified in the Alzheimer's disease NanoString panel

Gene	Log_2 Fold Change	P-Value	FDR
NDRG1	4.96	8.09E-10	6.16E-07
FAM107B	2.30	7.38E-09	2.81E-06
PDE1C	-1.72	2.09E-08	5.31E-06
EPAS1	1.96	6.61E-08	1.26E-05

ATG4C	-1.10	1.16E-06	1.77E-04
ALDH6A1	-1.40	1.75E-06	2.22E-04
PAK3	-1.42	2.07E-06	2.25E-04
TMCC1	-1.09	3.09E-06	2.94E-04
COPS4	-0.92	3.95E-06	3.35E-04
POGK	-1.00	4.54E-06	3.46E-04
SRI	-0.90	7.12E-06	4.93E-04
FRMD4B	2.11	9.79E-06	6.22E-04
NDE1	-0.85	1.48E-05	8.13E-04
DTD1	-1.05	1.49E-05	8.13E-04
DDX41	-0.98	2.01E-05	9.13E-04

Table S10. Tukey's multiple comparisons test for astrocytes for MTT reduction assay

Test Details:	Mean diff.	95.00% CI of diff.	Adjusted P Value
Control Normoxia vs. Infected Normoxia	0.01032	0.008422 to 0.01222	<0.0001
Control Normoxia vs. Control Hypoxia	-0.004392	-0.006290 to -0.002493	0.0008
Control Normoxia vs. Infected Hypoxia	0.01391	0.01201 to 0.01580	<0.0001
Infected Normoxia vs. Control Hypoxia	-0.01471	-0.01661 to -0.01281	<0.0001
Infected Normoxia vs. Infected Hypoxia	0.003585	0.001687 to 0.005484	0.0025
Control Hypoxia vs. Infected Hypoxia	0.0183	0.01640 to 0.02020	<0.0001

Table S11. Tukey's multiple comparisons test for microglia for MTT reduction assay

Test Details:	Mean diff.	95.00% CI of diff.	Adjusted P Value
Control Normoxia vs. Infected Normoxia	0.02397	0.01188 to 0.03607	0.0019
Control Normoxia vs. Control Hypoxia	-0.03115	-0.04324 to -0.01905	0.0005
Control Normoxia vs. Infected Hypoxia	0.02472	0.01263 to 0.03682	0.0016
Infected Normoxia vs. Control Hypoxia	-0.05512	-0.06722 to -0.04303	<0.0001
Infected Normoxia vs. Infected Hypoxia	0.0007502	-0.01134 to 0.01284	0.9961
Control Hypoxia vs. Infected Hypoxia	0.05587	0.04378 to 0.06797	<0.0001

Table S12. Tukey's multiple comparisons test for astrocytes for 2-NBDG uptake assay

Test Details:	Mean diff.	95.00% CI of diff.	Adjusted P Value
Control Normoxia vs. Infected Normoxia	209.6	86.52 to 332.7	0.0043
Control Normoxia vs. Control Hypoxia	-231.5	-354.6 to -108.3	0.0026
Control Normoxia vs. Infected Hypoxia	-87.22	-210.3 to 35.89	0.1664
Infected Normoxia vs. Control Hypoxia	-441.1	-564.2 to -318.0	<0.0001
Infected Normoxia vs. Infected Hypoxia	-296.8	-420.0 to -173.7	0.0007
Control Hypoxia vs. Infected Hypoxia	144.2	21.13 to 267.3	0.0257

Table S13. Tukey's multiple comparisons test for microglia for 2-NBDG uptake assay

Test Details:	Mean diff.	95.00% CI of diff.	Adjusted P Value
Control Normoxia vs. Infected Normoxia	-47.78	-183.4 to 87.82	0.638
Control Normoxia vs. Control Hypoxia	-292.5	-428.1 to -156.9	0.0012
Control Normoxia vs. Infected Hypoxia	-153	-288.6 to -17.35	0.0303
Infected Normoxia vs. Control Hypoxia	-244.7	-380.3 to -109.1	0.0032
Infected Normoxia vs. Infected Hypoxia	-105.2	-240.8 to 30.44	0.1257
Control Hypoxia vs. Infected Hypoxia	139.5	3.941 to 275.1	0.0445

Table S14. Tukey's multiple comparisons test for astrocytes for intracellular ATP assay

Test Details:	Mean diff.	95.00% CI of diff.	Adjusted P Value
Control Normoxia vs. Infected Normoxia	-124	-262.1 to 14.20	0.0758
Control Normoxia vs. Control Hypoxia	-113.6	-251.8 to 24.56	0.1034
Control Normoxia vs. Infected Hypoxia	-504.1	-642.3 to -366.0	<0.0001
Infected Normoxia vs. Control Hypoxia	10.36	-127.8 to 148.5	0.9932
Infected Normoxia vs. Infected Hypoxia	-380.2	-518.3 to -242.0	0.0003
Control Hypoxia vs. Infected Hypoxia	-390.5	-528.7 to -252.4	0.0003

Table S15. Tukey's multiple comparisons test for microglia for intracellular ATP assay

**Test Details:**	**Mean diff.**	**95.00% CI of diff.**	**Adjusted P Value**
Control Normoxia vs. Infected Normoxia	18.64	-25.02 to 62.30	0.5027
Control Normoxia vs. Control Hypoxia	-49.85	-93.51 to -6.197	0.0287
Control Normoxia vs. Infected Hypoxia	5.291	-38.37 to 48.95	0.9731
Infected Normoxia vs. Control Hypoxia	-68.5	-112.2 to -24.84	0.0065
Infected Normoxia vs. Infected Hypoxia	-13.35	-57.01 to 30.31	0.7246
Control Hypoxia vs. Infected Hypoxia	55.15	11.49 to 98.80	0.0183

Table S16. Tukey's multiple comparisons test for astrocyte extracellular Aβ40 levels

**Test Details:**	**Mean diff.**	**95.00% CI of diff.**	**Adjusted P Value**
Control Normoxia vs. Infected Normoxia	0.1634	0.04097 to 0.2859	0.0142
Control Normoxia vs. Control Hypoxia	0.1589	0.03648 to 0.2814	0.0162
Control Normoxia vs. Infected Hypoxia	0.2078	0.08538 to 0.3303	0.0044
Infected Normoxia vs. Control Hypoxia	-0.00449	-0.1270 to 0.1180	0.9992
Infected Normoxia vs. Infected Hypoxia	0.04441	-0.07805 to 0.1669	0.6188
Control Hypoxia vs. Infected Hypoxia	0.0489	-0.07356 to 0.1714	0.5516

Table S17. Tukey's multiple comparisons test for microglia extracellular Aβ40 levels

**Test Details:**	**Mean diff.**	**95.00% CI of diff.**	**Adjusted P Value**
Control Normoxia vs. Infected Normoxia	0.08832	0.06961 to 0.1070	<0.0001
Control Normoxia vs. Control Hypoxia	-0.1384	-0.1571 to -0.1197	<0.0001
Control Normoxia vs. Infected Hypoxia	-0.06472	-0.08343 to -0.04600	<0.0001
Infected Normoxia vs. Control Hypoxia	-0.2267	-0.2454 to -0.2080	<0.0001
Infected Normoxia vs. Infected Hypoxia	-0.153	-0.1718 to -0.1343	<0.0001
Control Hypoxia vs. Infected Hypoxia	0.07367	0.05495 to 0.09238	<0.0001

Table S18. Tukey's multiple comparisons test for astrocyte extracellular Aβ42 levels

Test Details:	Mean diff.	95.00% CI of diff.	Adjusted P Value
Control Normoxia vs. Infected Normoxia	-0.7377	-0.8891 to -0.5862	<0.0001
Control Normoxia vs. Control Hypoxia	-0.4899	-0.6414 to -0.3385	0.0001
Control Normoxia vs. Infected Hypoxia	-0.964	-1.116 to -0.8126	<0.0001
Infected Normoxia vs. Control Hypoxia	0.2477	0.09624 to 0.3992	0.0053
Infected Normoxia vs. Infected Hypoxia	-0.2264	-0.3779 to -0.07489	0.0083
Control Hypoxia vs. Infected Hypoxia	-0.4741	-0.6256 to -0.3226	0.0002

Table S19. Tukey's multiple comparisons test for microglia extracellular Aβ42 levels

Test Details:	Mean diff.	95.00% CI of diff.	Adjusted P Value
Control Normoxia vs. Infected Normoxia	-0.1334	-0.2387 to -0.02806	0.0181
Control Normoxia vs. Control Hypoxia	-0.06914	-0.1745 to 0.03619	0.2066
Control Normoxia vs. Infected Hypoxia	-0.5434	-0.6487 to -0.4380	<0.0001
Infected Normoxia vs. Control Hypoxia	0.06424	-0.04109 to 0.1696	0.2502
Infected Normoxia vs. Infected Hypoxia	-0.41	-0.5153 to -0.3046	<0.0001
Control Hypoxia vs. Infected Hypoxia	-0.4742	-0.5795 to -0.3689	<0.0001

Table S20. Tukey's multiple comparisons test for astrocyte extracellular Aβ42/Aβ40 ratio

Test Details:	Mean diff.	95.00% CI of diff.	Adjusted P Value
Control Normoxia vs. Infected Normoxia	-1.963	-2.384 to -1.543	<0.0001
Control Normoxia vs. Control Hypoxia	-1.328	-1.748 to -0.9072	0.0001
Control Normoxia vs. Infected Hypoxia	-2.895	-3.315 to -2.474	<0.0001
Infected Normoxia vs. Control Hypoxia	0.6353	0.2147 to 1.056	0.0078
Infected Normoxia vs. Infected Hypoxia	-0.9314	-1.352 to -0.5109	0.0011
Control Hypoxia vs. Infected Hypoxia	-1.567	-1.987 to -1.146	<0.0001

Table S21. Tukey's multiple comparisons test for microglia extracellular Aβ42/Aβ40 ratio

Test Details:	Mean diff.	95.00% CI of diff.	Adjusted P Value
Control Normoxia vs. Infected Normoxia	-2.882	-3.558 to -2.206	<0.0001
Control Normoxia vs. Control Hypoxia	-0.1055	-0.7814 to 0.5704	0.9459
Control Normoxia vs. Infected Hypoxia	-2.522	-3.198 to -1.846	<0.0001
Infected Normoxia vs. Control Hypoxia	2.776	2.101 to 3.452	<0.0001
Infected Normoxia vs. Infected Hypoxia	0.3599	-0.3160 to 1.036	0.3409
Control Hypoxia vs. Infected Hypoxia	-2.417	-3.093 to -1.741	<0.0001
